# The RADAR Study: Week 48 Safety and Efficacy of RAltegravir Combined with Boosted DARunavir Compared to Tenofovir/Emtricitabine Combined with Boosted Darunavir in Antiretroviral-Naive Patients. Impact on Bone Health

**DOI:** 10.1371/journal.pone.0106221

**Published:** 2014-08-29

**Authors:** Roger J. Bedimo, Henning Drechsler, Mamta Jain, James Cutrell, Song Zhang, Xilong Li, Irfan Farukhi, Rosinda Castanon, Pablo Tebas, Naim M. Maalouf

**Affiliations:** 1 Department of Medicine, VA North Texas Health Care System and University of Texas Southwestern Medical Center, Dallas, Texas, United States of America; 2 Department of Medicine, University of Texas Southwestern Medical Center, Dallas, Texas, United States of America; 3 Department of Medicine, VA North Texas Health Care System, Dallas, Texas, United States of America; 4 Department of Clinical Sciences, University of Texas Southwestern Medical Center, Dallas, Texas, United States of America; 5 Department of Nuclear Medicine, VA North Texas Health Care System, Dallas, Texas, United States of America; 6 Department of Medicine, University of Pennsylvania, Philadelphia, Pennsylvania, United States of America; University of New South Wales, Australia

## Abstract

**Background:**

NRTI-sparing regimens may avoid long-term mitochondrial, bone and renal toxicities and maintain viral suppression.

**Methods:**

In the RADAR study, 85 antiretroviral-naïve HIV-infected patients were randomized to receive either raltegravir (RAL) (n = 42) or tenofovir/emtricitabine (TDF/FTC) (n = 43), each with ritonavir-boosted darunavir (DRV/r). Virologic efficacy was assessed at weeks 24 and 48. Bone mineral density (BMD) was assessed by dual energy X-ray absorptiometry (DXA) scan at baseline and week 48, and bone turnover markers (BTM) assessed at weeks 0, 16 and 48.

**Results:**

Using an intention-to-treat analysis, 62.5% of RAL subjects and 83.7% of TDF/FTC subjects were responders (VL<48 copies/mL) at week 48 (p = 0.045; chi-square test). The proportions of patients achieving VL<200 copies/mL were similar: 72.5% and 86.0% (p = 0.175). Premature treatment discontinuation was the main cause for failure. No treatment-emergent resistance was observed. Changes from baseline in RAL vs. TDF/FTC for CD4^+^ (+199 vs. +216 cells/µL, p = 0.63), total cholesterol/HDL (−0.25 vs. −0.71 mg/dL (p = 0.270), and eGFR (−4.4 vs. −7.9 ml/min, p = 0.44) were comparable between groups. Changes in subtotal BMD to week 48 were: +9.2 with RAL vs. −7 g/cm^2^ with TDF/FTC (p = 0.002). Mean CTX changes were +0.04 vs. +0.24 ng/mL (p = 0.001), and mean P1NP changes were +3.59 vs. +30.09 ng/mL (p = 0.023). BTM changes at week 16 predicted change in BMD by week 48 (R = −0.394, p = 0.003 for CTX; and R = −0.477, p<0.001 for P1NP).

**Conclusion:**

The NRTI-sparing regimen RAL+DRV/r did not achieve similar week 48 virologic efficacy compared with TDF/FTC+DRV/r, but was better with regard to markers of bone health.

**Trial Registration:**

ClinicalTrials.gov NCT 00677300

## Introduction

All currently recommended regimens for antiretroviral-naive HIV-infected patients include tenofovir with emtricitabine (FTC) or lamivudine (3TC) in combination with either a non-nucleoside reverse transcriptase inhibitor (NNRTI), a boosted protease inhibitor (PI), or an integrase inhibitor (http://aidsinfo.nih.gov/guidelines). Although these regimens are potent and well tolerated, some concerns have emerged over the long-term bone and renal toxicities of tenofovir-containing regimens, particularly in the aging HIV-infected population, and those with significant comorbidities and increased fracture risk [1]. The primary alternative nucleoside abacavir (ABC) has been associated with hypersensitivity reactions and increased cardiovascular risk in some observational cohorts [2], [3]. These issues, together with concerns about lower virologic potency observed in a clinical trial [4] have tempered enthusiasm for abacavir use as first line therapy, except when combined with the new integrase inhibitor dolutegravir. This has led to an interest in designing studies to evaluate the safety and efficacy of nucleoside-sparing regimens. Regimens that combine a boosted PI with efavirenz are complex due to frequent pharmacokinetic interactions and associated with increased hyperlipidemia [5]. Given the potency of raltegravir (RAL) in naïve individuals [6], combining RAL with a boosted PI in HIV-1 became an obvious target. The first of such regimens to be evaluated in a longitudinal study was RAL with lopinavir/ritonavir (LPV/RTV), which was associated with similar virologic and immunologic efficacy as tenofovir/emtricitabine (TDF/FTC) and RAL for up to 96 weeks in the PROGRESS study [7], [8]. Following this, the SPARTAN study showed higher rates of hyperbilirubinemia and RAL resistance with the ATV/RTV + RAL regimen despite achieving similar virologic efficacy at 48 weeks as TDF/FTC + ATV/RTV [9]. Finally, a single-arm study (ACTG 5262) [10] demonstrated high rates of virologic failure and RAL resistance with a regimen of ritonavir-boosted darunavir (DRV/RTV) + RAL, mostly in patients with high baseline viremia. The absence of a comparator arm, however, precluded conclusive evaluation of the efficacy of DRV/RTV + RAL. This surprising lack of potency has become an anticipated observation of most non-NRTI-containing regimens.

The initiation of NRTI-containing antiretroviral therapy has been consistently associated with an initial decline in bone mineral density (BMD) that tends to stabilize over time, and these changes have been associated with a rapid increase in bone turnover markers (BTMs), which probably explain the decrease in BMD [11]–[16]. Compared with ABC, TDF is associated with greater increases in BTMs [17], [18]. HAART containing TDF has also demonstrated a faster decline in BMD than non-TDF-containing HAART [19], [20]. NRTI-sparing regimens may avoid the long-term adverse skeletal effects of NRTI. While patients on an NRTI-free regimen showed smaller decline in BMD than those on TDF-containing HAART in the PROGRESS study [7], [8], comparative data between NRTI-containing and NRTI-sparing regimens investigating the relationship between BTM and BMD changes are lacking.

We hereby report the results of the RADAR study, an open-label randomized, 48-week pilot study to evaluate the efficacy and safety of RAL + DRV/RTV compared with TDF/FTC + DRV/RTV in antiretroviral-naive subjects with HIV-1 RNA≥5,000 copies/mL.

## Materials and Methods

### Study design

The protocol for this trial and supporting CONSORT checklist are available as supporting information; see Checklist S1 and Protocol S1. The Institutional Review Board (IRB) of the VA North Texas Health Care System, Dallas, TX, approved this study, and an IRB-approved written informed consent was obtained from each participant. Patients were recruited from VANTHCS and Parkland Health and Hospital Systems. All study procedures were performed and data were collected at VANTHCS.

Study subjects were randomized in a 1∶1 ratio to receive either a fixed dose combination TDF/FTC (300/200 mg once daily) or RAL (400 mg twice daily), each in combination with DRV/RTV (800/100 mg once daily). The randomization scheme was generated by the Principal Investigator using the web site Randomization.com (http://www.randomization.com).

Patients were eligible if they were ≥18 years, antiretroviral naïve, had a plasma HIV-1 RNA concentration ≥5000 copies/mL, and a CD4 T cell count ≥100 cells/µL. Exclusion criteria were evidence of resistance to TDF, FTC or DRV. RAL resistance was not tested at baseline.

### Outcome measures and statistical methods

The primary outcome measure was virologic efficacy assessed by time to loss of virologic response until week 24. Secondary outcome measures included change from baseline in CD4 cell counts at weeks 24 & 48, the proportion of patients with HIV RNA<48 and <200 copies/mL at week 48, as well as safety endpoints including lipid profiles, BMD, BTMs, and inflammatory cytokines. For virologic efficacy analysis, patients were also stratified according to baseline viral load (VL)<100,000 or ≥100,000 copies/mL and baseline CD4 cell count (<200 or ≥200 cells/µL).

The planned sample size of 80 participants (40 per study arm) was increased to 85 to account for exclusion of enrolled participants who never started study treatment and never returned for follow-up after baseline evaluation.

### Virologic efficacy

Virologic efficacy was analyzed using the FDA time to loss of virologic response (TLOVR) algorithm, in which time of failure is defined as the earliest of any of the following events: death, permanent discontinuation of the study drug, loss to follow-up, or plasma HIV-1 RNA concentrations >48 copies/ml obtained at two consecutive visits or one value >48 copies/ml followed by permanent discontinuation of the study drug or loss to follow-up. This was the standard antiretroviral study analysis when the study was conceived, and has been substituted recently by the snapshot analysis [21]. All subjects who were randomized and received at least one dose of the study drugs were included in the analysis (intention to treat, ITT analysis). Another analysis was performed censoring participants lost to follow-up or who died without previously meeting the definition of virologic failure (“on treatment”, OT analysis). We repeated the analyses using the newer FDA Snapshot methodology, where participants with viral suppression at week 48 were classified as successes. Participants missing HIV RNA data at week 48 analysis, or who discontinued study drug before week 48 were classified as failures.

### Bone health assessment

All patients underwent whole-body DXA scanning (Hologic, QDR 4500A) at baseline (week 0) and at week 48 to assess total body and subtotal (total minus head) BMD, as well as fat and lean body mass. Fasting plasma samples were collected and frozen at −70°C. Markers of inflammation and bone turnover were measured at weeks 0, 16, and 48. All assays were performed blinded to treatment group and BMD measurements. Inflammatory markers measured were: soluble tumor necrosis factor (TNF)-α, interleukin 1b (IL1-B), and IL-6 (Meso Scale Discovery, Rockville, MD). BTMs measured included C-terminal telopeptide of type 1 collagen (CTX, marker of bone resorption), and N-terminal type 1 procollagen (P1NP, marker of bone formation). CTX was measured using a luminometric assay on Elecsys 2010 (Hoffmann-La Roche); reference range: men 0.158–0.584 ng/mL, pre-menopausal women 0.162–0.573 ng/ml, post-menopausal women 0.330–1.008 ng/ml; Inter-assay coefficient of variance <8.9%. P1NP was measured with radioimmunoassay (UniQ P1NP RIA kit; Orion Diagnostica) reference range 25.91–132.5 ng/mL; Inter-assay coefficient of variance <12.4%.

### Statistical Analysis

Between-group comparisons for continuous variables were carried out using t-test if normally distributed and non-parametric Wilcoxon rank sum test if not normally distributed. For categorical variables, we used chi-square test or Fisher’s exact test. Finally, correlations were assessed using Spearman correlation coefficient. All analyses were conducted using PASW (SPSS) version 18 (IBM, Armonk, N.Y.).

## Results

### Study participants

**Figure 1:**
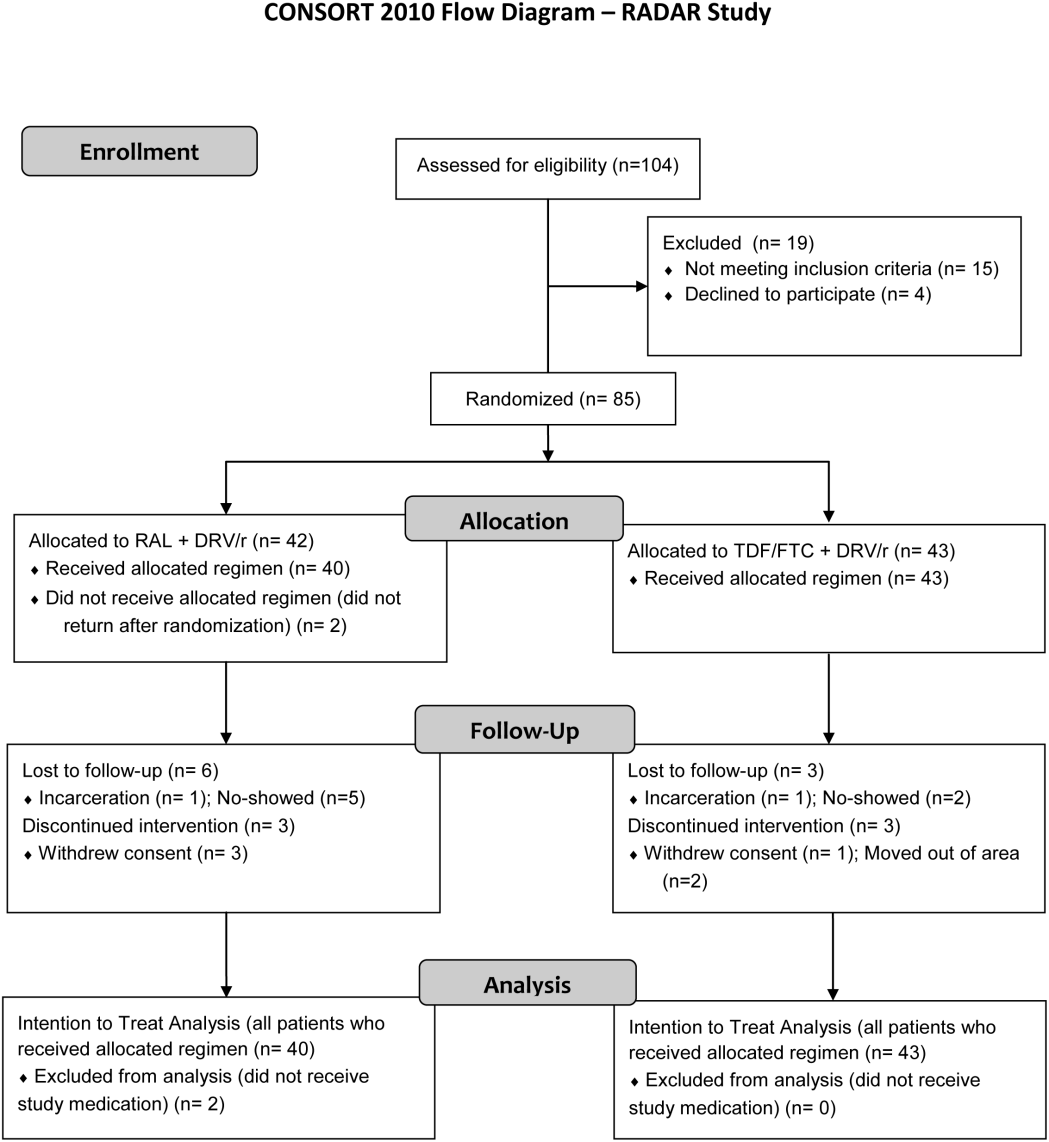
CONSORT Flow Diagram.

Patients were recruited between February 2009 and November 2011. A total of 85 subjects were randomized: 42 in the RAL group and 43 in the TDF/FTC. Two patients did not return following randomization, and 83 patients (40 RAL group and 43 TDF/FTC group) received at least one dose of study medication and were included in the ITT analysis. Of those, 4 others did not return for their first follow-up visit. The remaining 79 patients (39 RAL and 40 TDF/FTC) were included in the OT analysis (Figure 1). Demographic and baseline characteristics are presented in table 1, and mean baseline laboratory values are presented in table 2. The proportion of patients with baseline plasma HIV-1 RNA≥100,000 copies/mL was similar in both groups (13/40 in RAL and 19/43 in TDF/FTC).

**Table 1:** Demographics and Baseline Characteristics.

Median (± IQR or %)	DRV/r + RAL	DRV/r + TDF/FTC
n =	40	43
Age, years	43.8 (33.3–51.0)	39.1 (26.2–46.1)
Race/Ethnicity		
African American	18 (45%)	22 (51%)
European American	12 (29%)	10 (23%)
Hispanic	10 (26%)	11 (26%)
Male Gender	36 (90%)	41 (95%)
HCV-antibody positive	5 (13%)	5 (12%)
CD4 count at baseline (cells/µL)	249 (164–432)	201 (67–358)
HIV Viral Load (log copies/mL)	4.69 (4.17–5.21)	4.92 (4.29–5.40)

**Table 2:** Lipid parameters and Renal Function: Baseline values and changes at week 48.

	Mean Baseline Values		Mean Changes From Baseline (95% Confidence Interval)		
	DRV/r + RAL	DRV/r + TDF/FTC	DRV/r + RAL	DRV/r+ TDF/FTC	P-value
Total Cholesterol (mg/dL)	159	149	+23.3 (+14.5– +32.2)	+6.5 (−1.4– +14.4)	0.003
LDL-Cholesterol (mg/dL)	94	88	+11.2 (+2.6– +19.8)	+3.3 (−4.6– +11.2)	0.097
HDL-Cholesterol (mg/dL)	40	34	+4.8 (−1.1– +10.8)	+7.2 (+3.7– +10.7)	0.796
Triglycerides(mg/dL)	131	182	+21.2 (−6.9– +49.2)	−38.1 (−113.6– +37.4)	0.156
Total Cholesterol/HDL	4.59	4.78	−0.25 (−0.83– +0.34)	−0.71 (−1.12– +0.29)	0.270
Estimated GFR by CKD-EPI formula(ml/min)	104	110	−4.4 (+3.2– −11.6)	−7.9 (−2.5– −13.7)	0.44

### Virologic efficacy at week 24

In the primary analysis using the FDA TLOVR analysis, 75% (30/40) of the RAL + DRV/RTV patients and 76.7% (33/43) of the TDF/FTC + DRV/RTV has plasma HIV-1 RNA<48 copies/mL at week 24; difference: −1.7% (95% CI: −20.2% to +16.7%); p = 0.853. Percent virologic responses were the same using the FDA Snapshot analysis.

### Virologic efficacy at week 48

**Figure 2:**
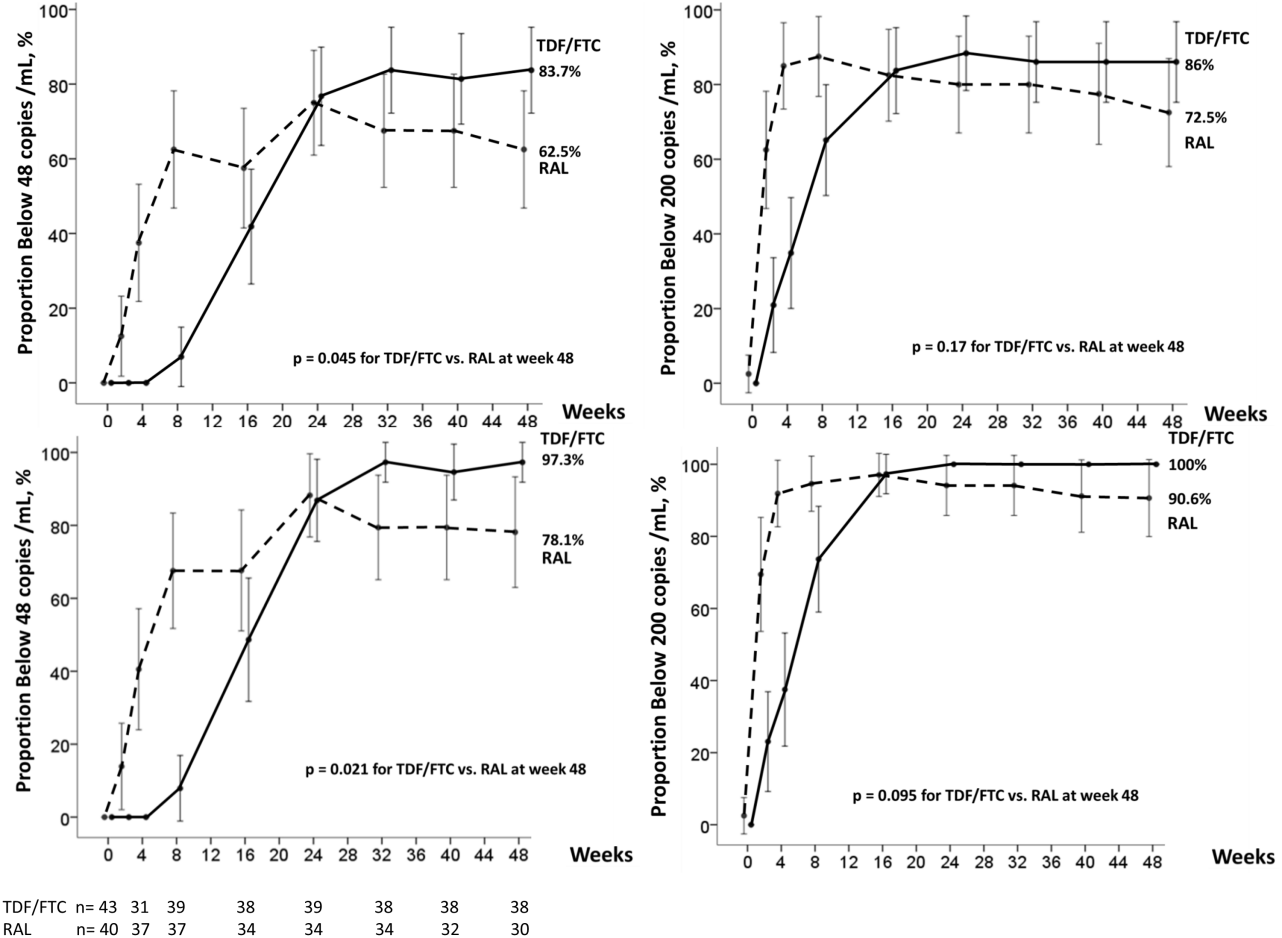
Proportion of subjects with plasma HIV-1 RNA<48 copies/mL and <200 copies/mL using the Intention-to-Treat Analysis (top) and the On-Treatment-Analysis (bottom, with number of patients indicated).

Using the ITT TLOVR analysis until week 48, mean time to loss of virologic response (>48 copies/mL) was 36.3 weeks in the RAL arm and 42.1 weeks in the TDF/FTC arm. Percent virologic responders were 60% (24/40) and 83.7% (36/43); difference: −23.7% (95% CI: −42.9% to −5.0%); p = 0.016. Using the FDA snapshot analysis, at 48 weeks, 62.5% of DRV/r +RAL subjects and 83.7% of DRV/r + TDF/FTC subjects were responders; difference: −21.2% (95% CI: −39.8% to −2.6%); p = 0.045; chi-square test) (Figure 2A). There was no statistically significant difference in the proportions of patients achieving VL<200 copies/mL: 72.5% (29/40) and 86.0% (37/43) respectively; difference: −13.5% (95% CI: −30.8% to −3.7%); p = 0.175 (Figure 2B). Patients on the RAL arm achieved more rapid virologic suppression than participants in the TDF/FTC arm; of the patients remaining in the study at week 48, 27/34 (79%) in the RAL arm had already achieved a viral load <48 copies/mL at week 16 compared to 19/37 (51%) patients in the TDF/FTC arm (Figure 2A).

In the OT analysis, censoring follow-up if a participant was lost to follow-up without previously meeting the definition of virologic failure, the percent responders at week 48 (VL<48 copies/mL) was 78.1% in the RAL and 97.3% in the TDF/FTC groups (p = 0.021) (figure 2C). The proportions of patients achieving VL<200 copies/mL were 90.6% and 100.0% respectively (p = 0.095) (figure 2D).

Viral load change from baseline was −2.78 log_10_ for RAL patients and −3.08 log_10_ for TDF/FTC patients (p = 0.175). In subjects with baseline plasma HIV-1 RNA≥100,000 copies/ml, the RAL and TDF/FTC groups had comparable proportions of non-responders (7/14 vs. 6/19, p = 0.165). Evaluation of treatment adherence by questionnaire during study visits did not show significant differences in reported adherence between the two regimens.

### Treatment Discontinuations

During the study, 9 (22.5%) RAL subjects and 6 (14%) TDF/FTC subjects discontinued study or study drugs (table 3) (p = 0.468; chi-square test). All but two discontinuations were due to loss to follow-up. Only two patients in the RAL arm and none of the TDF/FTC patients had a VL>200 copies/mL at the time of study discontinuation. Three patients in the RAL group, and none in the TDF/FTC group, who completed the study had VL>48 at week 48 (Table 3). Resistance testing showed no treatment-emergent resistance-associated mutations.

**Table 3:** Virologic Outcome at Week 48 - FDA Snapshot Analysis.

	RAL + DRV/rN = 40	TDF/FTC + DRV/rN = 43
HIV-RNA<48 copies/mL	25 (62.5%)	36 (83.7%)
HIV-RNA**≥**48 copies/mL	3 (7.5%)	0 (0%)
No Virologic Data at Week 48Reasons:	12 (30.0%)	7 (16.3%)
• Discontinued study/study drugs due to AE or death	0 (0%)	0 (0%)
• Discontinued study/study drugs for other reasons(loss to f/u; withdrew consent)	9 (22.5%)	6 (14.0%)
• On study but missing data at week 48	3 (7.5%)	1 (2.3%)

### Immunologic response

The median (IQR) CD4 T cell count changes from baseline to week 48 were 167 (120–281) cells/µL in the RAL group and 207 (80–330) cells/µL in the TDF/FTC group. The mean changes (95% CI) were 199 (150–248) cells/µL in the DRV/r + RAL group and 216 (170–273) cells/µL in the DRV/r + TDF/FTC group (p = 0.63) (Figure S1).

### Adverse Events

Seven patients (5 in the RAL and 2 in the TDF/FTC groups) reported at least one grade 3 (severe) or higher clinical or laboratory adverse events, one of which was classified as possibly related to study drugs (elevated liver enzymes). No events were considered probably or definitely related to study treatment. No deaths were observed during the follow-up period.

### Lipid Profile and Renal Function

Mean increase in serum total cholesterol (TC) from baseline to week 48 was greater in the RAL group: +23.3 mg/dL; (95% CI: +14.5– +32.2) than in the TDF/FTC group: +6.5 mg/dL (−1.4– +14.4) (p = 0.003, Table 2). However, there were comparable mean changes between the RAL and the TDF/FTC groups in other lipid parameters. Serum low-density lipoprotein (LDL)-cholesterol: +11.2 mg/dL (+2.6– +19.8) vs. +3.3 mg/dL (−4.6– +11.2) (p = 0.097); serum high-density lipoprotein (HDL)-cholesterol: +4.8 mg/dL (−1.1– +10.8) vs. +7.2 mg/dL (+3.7– +10.7) (p = 0.796); serum triglyceride: +21.2 mg/dL (−6.9– +49.2) vs. −38.1 mg/dL (−113.6– +37.4) (p = 0.156); and TC/HDL: −0.25 (−0.83– +0.34) vs. −0.71 (−1.12– +0.29) (p = 0.270). The change in estimated GFR was also similar between the two groups: 4.4 ml/min (+3.2– −11.6) in RAL vs. −7.9 ml/min (−2.5– −13.7) in TDF/FTC (p = 0.44) (Table 2).

### Body fat and bone mineral density

Whole-body DXA analyses were utilized to compare the changes in body fat and BMD between the regimens. At baseline, fat in the arms, legs, and trunk, and BMD were similar between groups.

Changes from baseline to week 48 showed statistically significant differences between the two treatment arms for subtotal and total BMD, but not for fat or lean body mass. Changes in subtotal BMD from baseline to week 48 were +9.2 g/cm^2^ in the RAL group and −7 g/cm^2^ in the TDF/FTC group (p = 0.002), while total BMD changes were +11.3 g/cm^2^ and −6.9 g/cm^2^ respectively (p = 0.013). Changes in total fat were +3.13 kg in the RAL group and +1.80 kg in the TDF/FTC group (p = 0.430) and changes in total lean body mass were +1.48 kg and +0.60 kg in the RAL and TDF/FTC groups, respectively (p = 0.084).

### Markers of bone turnover

**Figure 3:**
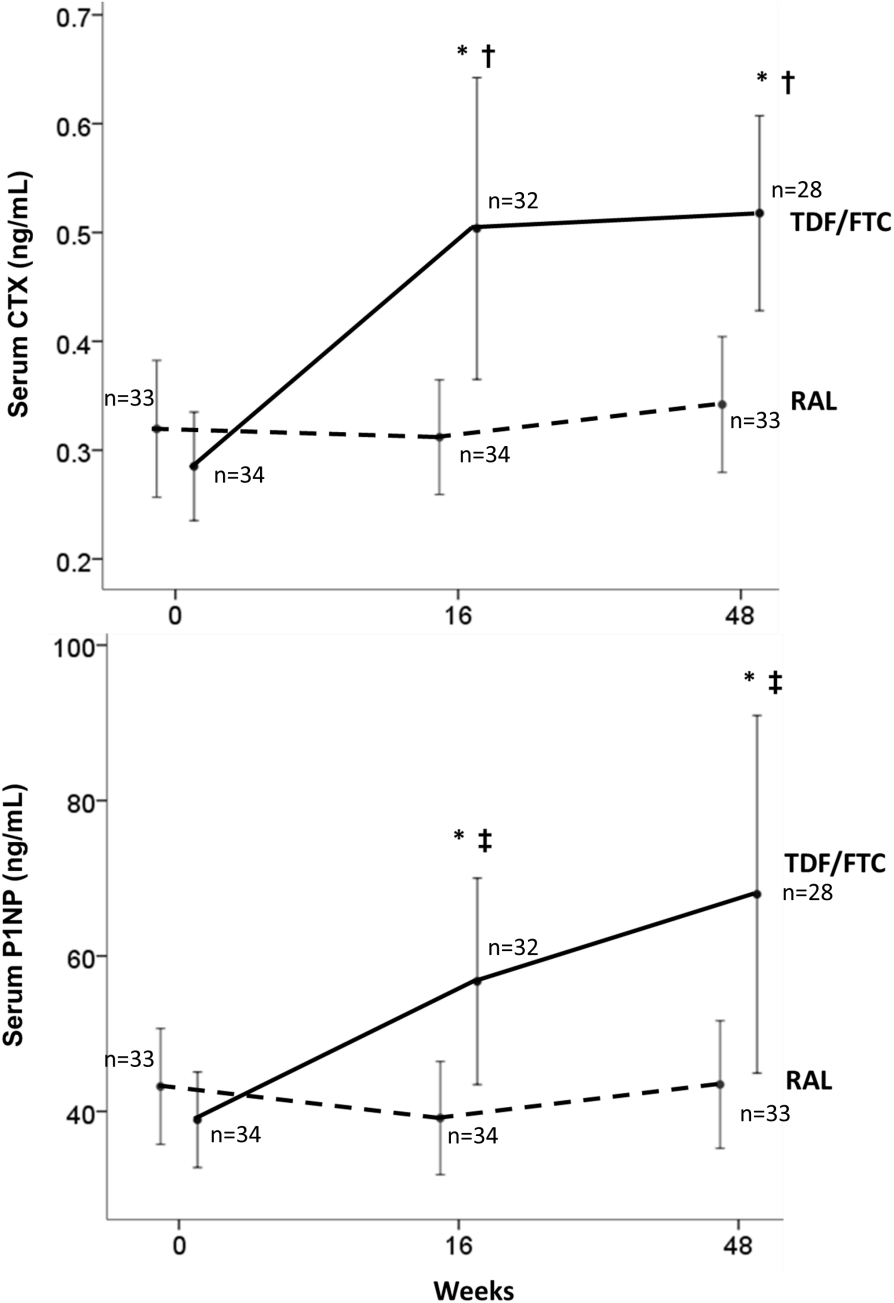
Changes in bone turnover markers from baseline to week 48 (number of patients indicated). Top: Serum Terminal Telopeptide (CTX). †p<0.01 for TDF/FTC vs. RAL groups at specific time point *p<0.01 for TDF/FTC group versus baseline. Bottom: Serum Procollagen type 1 N-terminal propeptide (P1NP, bottom). ‡p<0.05 for TDF/FTC vs. RAL groups at specific time point *p<0.01 for TDF/FTC group versus baseline.

Mean CTX and P1NP were similar between the two groups at baseline (0.32 vs. 0.29 ng/mL for CTX, p = 0.36, and 43.2 vs. 40.9 ng/mL for P1NP, p = 0.38). Mean CTX changes from baseline to week 48 were +0.04 ng/mL (CI: −0.03– +0.11) in the RAL group and +0.24 ng/mL (CI: +0.17– +0.32) in the TDF/FTC group (p = 0.001). Mean P1NP changes from baseline to week 48 were +3.59 ng/mL (CI: −3.89– +11.06) in the RAL group and +30.09 ng/mL (+9.75– +50.42) in the TDF/FTC group (p = 0.023). (Figure 3A and 3B).

Both CTX and P1NP changes occurred early in the TDF/FTC group and were significant at week 16+0.23 ng/mL (+0.12– +0.35; p<0.001) for CTX and +18.92 ng/mL (+7.19– +30.64; p = 0.001) for P1NP. However, CTX appeared to increase earlier, reaching the maximum level at week 16 (mean: 0.50 ng/mL) and remaining stable to week 48 (mean: 0.52 ng/mL). On the other hand, P1NP levels increased more gradually, and appeared to continue to increase from week 16 to week 48 (+11.2 ng/mL, p = 0.071).

**Figure 4:**
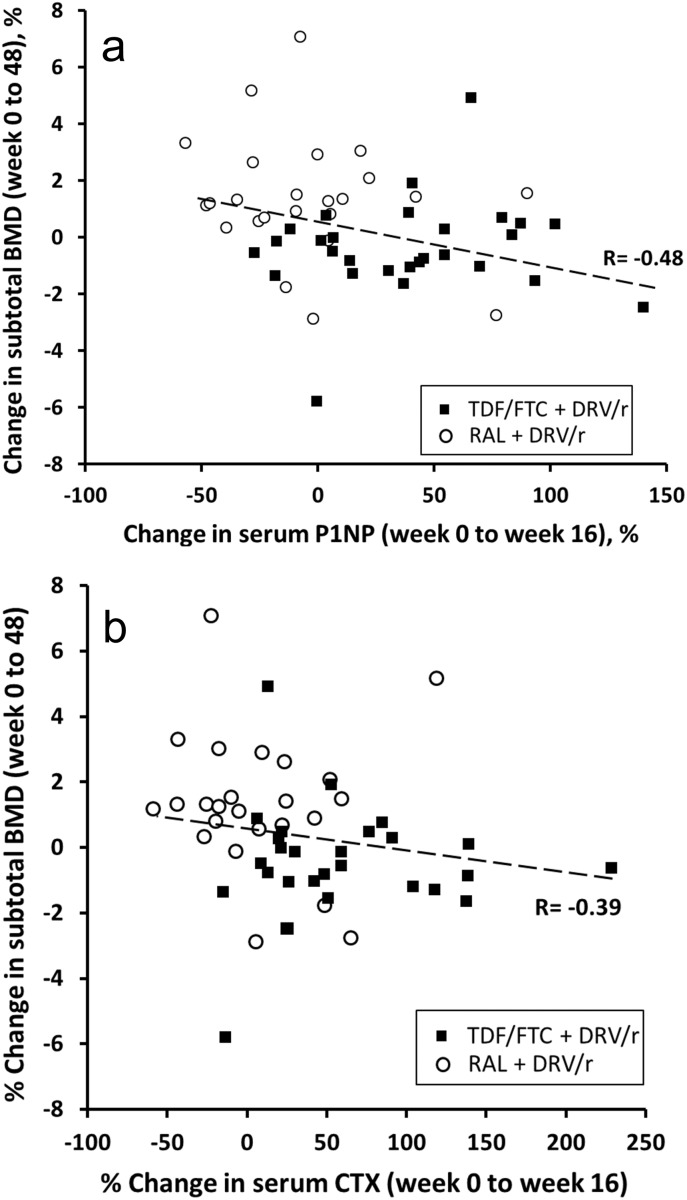
Correlation between early changes in bone turnover markers (week 16 vs. week 0) and change in BMD by week 48. Figure 4A: Correlation for serum P1NP. Figure 4B: Correlation for serum CTX.

Early changes in CTX and P1NP at week 16 were correlated to subsequent changes in BMD at week 48 (Figure 4A and 4B).

### Markers of inflammation: Correlation with Bone Turnover Markers

Both regimens lead to similar changes in markers of inflammation at week 16 and 48.

There were significant decreases in mean TNF-α levels from baseline to week 16 (−2.75 and −1.93 pg/mL in the RAL and TDF/FTC groups, respectively, p = 0.177). Changes from baseline to week 48 were −2.48 and −2.85 pg/mL; p = 0.465). TNF-α changes (baseline vs. week 48) were not significantly correlated to changes in CTX or PN1P in either the RAL group (TNF-α vs. CTX: r = −0.19, p = 0.35; TNF-α vs. P1NP: r = 0.13, p = 0.55), or in the TDF/FTC group (TNF-α vs. CTX: r = 0.33, p = 0.08; TNF-α vs. P1NP: r = −0.14, p = 0.94). Similarly, there were no differences between groups in the changes in IL-1b (+0.55 vs. +0.67 pg/mL; p = 0.866), or IL-6 (+0.95 vs. −1.00 pg/mL; p = 0.259). Changes in IL-1b or IL-6 levels were also not correlated with changes in BTMs (data not shown).

## Discussion

In this open-label randomized 48-week study in antiretroviral-naive HIV-infected subjects, we found the NRTI-sparing regimen RAL+ DRV/RTV to be virologically inferior to TDF/FTC + DRV/RTV, despite similar virologic response at week 24. Both regimens led to similar changes in inflammatory markers over time but had distinctly different effects on bone turnover markers and BMD. TDF/FTC use led to an early increase in bone turnover markers, which was associated with a decline in BMD, whereas RAL use did not significantly impact bone health.

Our study was originally designed with a non-inferiority comparison in mind. We accept, however, that our sample size was inadequate to have had any reasonable power to establish non-inferiority using typical non-inferiority deltas of 12% or 15%. Our results, though, show fewer treatment failures for TDF/FTC + DRV/RTV at week 48, albeit the majority of these failures were due to lost to follow-up and treatment discontinuation rather than true virologic failure.

The virologic response rate to RAL + DRV/RTV was similar to that observed in ACTG 5262 [10] (Table S1). All but three treatment failures in the RAL group resulted from loss to follow-up or withdrawal of consent. While the twice-daily administration for RAL in the RAL + DRV/r regimen could have resulted in a lower acceptance rate and sub-optimal adherence, there was no significant difference in self-reported medication adherence between the two regimens in our study. The recently completed ANRS 143/NEAT 001 study (NCT: NCT01066962) – with similar design as RADAR but much larger sample size – will likely contribute to more definitively answering the question of whether a difference in treatment efficacy exists.

The two regimens achieved comparable immunologic response, but patients in the TDF/FTC arm had smaller increases in total cholesterol. This is in line with other studies confirming a lower impact on serum cholesterol – or possibly a cholesterol-lowering property – associated with TDF [22]. Except for TC, there were no statistically significant differences in lipid changes from baseline between the two groups. There was also no statistically significant difference in the mean changes in serum creatinine and eGFR between groups.

Similar to the PROGRESS study, changes in BMD were significantly worse in the TDF arm than in the NRTI-sparing regimen. Furthermore, patients in the TDF arm had significant sustained increases in BTMs at week 16 and 48 while RAL patients did not. Changes in BTMs at week 16 were predictive of BMD decline at week 48. If confirmed in additional studies, early changes in BTMs may be useful to predict long-term BMD decline and allow providers to make regimen changes in a timely manner and also simplify the monitoring of bone safety in large antiretroviral trials. As the standard of care is DXA scans, the enrollment in studies monitoring BMD becomes more complex and expensive since sites need access to a good radiology center and central reading, biasing enrollment towards tertiary referral centers and a relatively small subset of the participants. If the observation that changes in BTM predict changes in BMD is confirmed – as appears to be the case in a recently published study [23] – the monitoring of trials could be done by evaluation of BTM, which would allow enrollment of a large proportion of participants, reduce selection bias, and increase generalizability. Bone resorption (CTX) appears to increase much earlier than formation (P1NP), suggesting the latter is compensatory. This appears to confirm the hypothesis of a “catabolic window” [16] following initiation of HAART that might lead to decreased BMD. The STEAL and ASSERT studies [17], [18] have previously shown a lower increases in BTMs with ABC/3TC compared to TDF/FTC, each in combination with boosted protease inhibitors. Furthermore, there were greater increases in the resorption marker CTX among patients receiving ATV/RTV vs. Efavirenz (EFV) in combination with TDF/FTC [24]. In the only previous evaluation of BTMs in an NRTI-free regimen, patients randomized to the nevirapine (NVP) arm still had significant elevations in BTMs from baseline, albeit lower than those randomized to zidovudine/lamivudine (AZT/3TC) [16]. To our knowledge, this is the first report of an antiretroviral regimen without significant changes in BTMs at week 48 suggesting that NRTI-sparing regimens could potentially be bone neutral. This finding also suggests that the initial bone loss associated with antiretroviral treatment most likely represents a direct effect of medications, rather than a general phenomenon of immune reconstitution.

It is widely assumed that increased HIV-associated inflammation and immune activation are the drivers of many non-AIDS complications. Accordingly, bone demineralization has been postulated to result from enhanced inflammation resulting in excessive stimulation of osteoclastogenesis leading to a net surplus of bone resorption [25]. However, as illustrated by our study, the pathogenesis of osteoporosis is likely more complex as both regimens were associated with similar changes in inflammatory markers over time but had distinctly different effects on viral kinetics, BTMs, and BMD.

There are several limitations to our study that call for caution in interpreting the data presented. First, our study had a relative sample size that only allowed for the detection of larger differences between the arms. Second, we did use total and subtotal BMD, which are less precise than site-specific BMD. Also, while the measurements of bone turnover markers have been presented in HIV-infected patients in previous studies, reference ranges in this population are still unclear. Finally, the overwhelmingly male composition of the study population might limit its generalizability. Nonetheless, our findings appear to be in line with previous studies evaluating the nucleoside-free regimens such as ACTG 5262 (Table S1).

In summary, while not achieving a comparable virologic response, the NRTI-sparing regimen RAL + DRV/RTV was significantly more bone neutral than standard HAART with TDF/FTC + DRV/RTV. As bone health becomes more relevant in an aging HIV population, this potential benefit of NRTI-sparing regimens deserves closer examination.

### Previous Presentations of the Data

Bedimo R, Drechsler H, Cutrell J, Jain M, Tebas P, and Maalouf N. RADAR Study: Week 48 Safety and Efficacy of Raltegravir combined with boosted Darunavir Compared to Tenofovir/Emtricitabine combined with boosted Darunavir in antiretroviral-naive patients. Impact on bone health. 7*th IAS Conference on HIV Pathogenesis, Treatment and Prevention*. Abstract WEPE512. Kuala Lumpur, Malaysia; June 30–July 3, 2013.

## Supporting Information

Figure S1 **Mean CD4 count change from baseline to week 48 (cell/µL).**
(TIF)

Table S1 Comparison of RADAR virologic and immunologic results with those of A5262 and PROGRESS studies.
(DOC)

Checklist S1 **RADAR study CONSORT checklist.**
(DOC)

Consent S1 **RADAR study consent form – Initial.**
(PDF)

Consent S2 **RADAR study consent form – First Revision.**
(PDF)

Consent S3 **RADAR study consent form – Second Revision.**
(PDF)

Protocol S1 **RADAR study protocol (abbreviated) – Initial.**
(PDF)

Protocol S2 **RADAR study protocol – Revised.**
(PDF)
