# Supplementary material for: The RADAR Study: Week 48 Safety and Efficacy of RAltegravir Combined with Boosted DARunavir Compared to Tenofovir/Emtricitabine Combined with Boosted Darunavir in Antiretroviral-Naive Patients. Impact on Bone Health
Source: PLoS One. 2014 Aug 29;9(8):e106221. doi: 10.1371/journal.pone.0106221 (PMC4149560; doi:10.1371/journal.pone.0106221)
Supplement: Table S1 — Comparison of RADAR virologic and immunologic results with those of A5262 and PROGRESS studies. (DOC) [file pone.0106221.s002.doc]

**Table S1.** Comparison of RADAR virologic and immunologic results with those of A5262 and PROGRESS studies.

| Variable | A5262 | RADAR | | PROGRESS | |
| --- | --- | --- | --- | --- | --- |
|  | DRV/r+ RAL (n=112) | DRV/r+ RAL (n=40) | DRV/r+ TDF/FTC (n=43) | LPV/r+ RAL (n=101) | LPV/r+ TDF/FTC (n=105) |
| Mean BL VL log_10_  (SD or range) | 4.83  (0.6) | 4.62 (4.41-4.82) | 4.85 (4.6-5.1) | 4.24 (2.0-6.0) | 4.25 (2.7–6.0) |
| Median BL CD4, cells/μL (IQR) | 271  (107-419) | 246  (164–432) | 201 (67–358) | 289 (SD: 149) | 298 (SD: 167) |
| VL<200(CI) NC=F | 73% (65-81) | 73% (58-86) | 86% (75-96) |  |  |
| VL<48 (CI) NC=F | 61% (52-70) | 63% (48-78) | 84% (72-94) | 81% | 86% |
| Median ∆CD4  cells/μL (IQR) | 200  (114-318) | 167 (120-281) | 207 (80-330) | 215 | 245 |

VL: Viral load. SD: Standard Deviation. CI: 95% confidence intervals. IQR: Interquartile Range.

The NRTI-free regimens are shaded.
