# Supplementary material for: The RADAR Study: Week 48 Safety and Efficacy of RAltegravir Combined with Boosted DARunavir Compared to Tenofovir/Emtricitabine Combined with Boosted Darunavir in Antiretroviral-Naive Patients. Impact on Bone Health
Source: PLoS One. 2014 Aug 29;9(8):e106221. doi: 10.1371/journal.pone.0106221 (PMC4149560; doi:10.1371/journal.pone.0106221)
Supplement: Consent S3 — RADAR study consent form – Second Revision. (PDF) [file pone.0106221.s006.pdf]

Department of Veterans Affairs

Dallas VA Medical Center Research Consent Form

Subject Name: ______________________ Date: ____________

Title of Study: Evaluation of Safety & Efficacy of Raltegravir/Darunavir Combination in Antiretroviral-Naïve Patients, #07-124

Principal Investigator: Roger Bedimo, M.D.

Co-Investigator(s): Henning Drechsler, M.D.; Teresa Moore, PA-C; Diana L. Turner, PA-C; Irfan Farukhi, M.D.

Study Coordinators: Holly Wise and Joyce Ghormley

Before agreeing to take part in this research study, it is important that you <u>read and understand the proposed research explained below</u>. It describes the procedures, benefits, risks and discomforts of the study. It also describes other treatments that are open to you and your right to withdraw from the study at any time. It is important for you to understand that no promises can be made about the results of the study.

1. WHAT IS THIS RESEARCH STUDY ABOUT?

Raltegravir is a leading candidate in a new class of antiretroviral medications called integrase inhibitors. Inhibition of integrase prevents the HIV DNA virus from entering into the human DNA genome, thus blocking the ability of HIV to reproduce and spread in the body. It is taken orally twice a day (every twelve hours) and does not require the addition of low-dose Ritonavir to achieve maximum beneficial effect.

Raltegravir has been shown to have excellent virologic effectiveness in patients who have not yet taken antiretrovirals (treatment naïve) and in heavily treatment experienced patients. It has also been shown to have unusually rapid virologic response. This result might be excellent in delaying viral resistance in naïve patients.

Darunavir is the newest drug in the class of protease inhibitors. It prevents the virus from assembling out of the infected cell and propagating the infection to other cells. It is taken orally once a day together with low-dose Ritonavir for patients who have not been previously on treatment. Darunavir has been shown to very potent and is currently approved in the treatment-experienced patients.

SUBJECTS IDENTIFICATION (I.D., plate or give name-last, first, middle)

Subject Name: ________________ Date: ________
Title of Study: Evaluation of Safety & Efficacy of Raltegravir/Darunavir Combination in Antiretroviral-Naïve Patients, #07-124
Principal Investigator: Roger Bedimo, M.D.
Co-Investigator(s): Henning Drechsler, M.D.; Teresa Moore, PA-C; Diana L. Turner, PA-C; Irfan Farukhi, M.D.

Study Coordinators: Holly Wise and Joyce Ghormley

Recent studies have also shown it to have excellent virologic effectiveness in patients who have not yet taken antiretrovirals (treatment naïve), with fewer side effects than other drugs of its class.

A combination of Raltegravir and Darunavir in treatment naïve patients has the potential of not only being very efficacious, but also to limit drug exposure and toxicity.

You have been asked to take part in this research study because you are an HIV positive individual and have not had any antiretroviral treatment. The supporters of this protocol are Merck & Co., Inc., North Wales, PA, and Tibotec Therapeutics, Bridgewater, NJ.

The enrollment goal for this study is 80 patients and the duration should last 48 months.

2. WHAT WILL HAPPEN DURING THE STUDY?

This is a randomized (like flipping a coin), open-label study to determine the safety and effectiveness of Raltegravir and Darunavir in treatment naïve patients.

This study consists of a screening period and a treatment period lasting up to 48 months. During the screening visit, the study doctor will check if you meet all the conditions to enter the study. Therefore, you will be asked some questions about your medical history, current medical condition and the medication you are taking. Participants will be randomized into two different treatment groups:

1. **Group A:** will receive Raltegravir (400mg twice daily) + Ritonavir-boosted (100mg once daily) Darunavir (800mg once daily)
2. **Group B:** will receive Tenofovir (300mg once daily) + Emtricitabine (200mg once daily) + Ritonavir-boosted (100mg once daily) Darunavir (800mg once daily)

If you participate in this study, you will be asked to return to your study doctor for regular visits for medical check-ups, study related evaluations, and/or dispensing and/or returning the study medication.

You will be asked to return to the clinic at weeks 2, 4, 8, and every 8 weeks thereafter while on treatment during this study. At these visits you will get a medical examination and blood will be taken. For women who can get pregnant, a urine dipstick pregnancy test will be taken at each visit.

At the end of the study, you will be asked to return to the clinic for a final/withdrawal visit. At this visit, a medical examination and blood will be taken. For women who can get pregnant, a urine pregnancy test will be performed.

Blood Sampling

All blood samples will be collected for routine safety testing by a local laboratory. Your immune function (CD4 cell count), your plasma viral load (the amount of HIV virus present in your blood) and your liver function.

The amount of blood taken per visit will be approximately 5 teaspoons. The local laboratory identified by the study physician will analyze all blood tests. No samples are collected by or transferred to the sponsor.

Instructions for Medication Intake

You will receive your study medication according to your treatment group at every visit during the treatment phase. You will also be asked to return all used and unused bottles at every study visit. During the study you will be asked to take your study medication as requested by your study doctor. You will also be instructed on how to report any side effects you may experience during this study.

You will be randomized to one the following treatment groups:

- Group A: Take Raltegravir 400mg twice daily, Darunavir 800mg once daily plus Ritonavir 100mg once daily (taken with food).

- Group B: Take Tenofovir 300mg once daily, Emtricitabine 200mg once daily, Darunavir 800mg once daily plus Ritonavir 100mg once daily (taken with food).

Please ask your study doctor what to do in case you miss a dose of the other HIV drugs you are taking with Raltegravir. Report all missed doses to the study coordinator and/or study doctor at your next visit.

3. WHAT ARE MY RISKS?

By participating in this study, your condition will be closely monitored. It is possible that by participating in this study your condition may improve, and that this study may be helpful in developing a new therapy for others with similar illnesses. However, you may not benefit from participation in this study.

Medication Side Effects

Raltegravir

The most common side effects (occurred in at least 5 out of 100 patients in any of the studies) that were judged related to Raltegravir, given along with other HIV drugs are:

• Nausea	• Difficulty sleeping (insomnia)
• Headache	• Abnormal dreams
• Dizziness	• Intestinal gas (flatulence)
• Diarrhea	

♦ Less common side effects (occurred in more than 2 but less than 5 out of 100 patients in any of the studies) that were judged related to Raltegravir, given along with other HIV drugs are:

• Itching (pruritus)	• Sleepiness (somnolence)

Subject Name: ______________________ Date: ____________

Title of Study: Evaluation of Safety & Efficacy of Raltegravir/Darunavir Combination in Antiretroviral-Naïve Patients, #07-124

Principal Investigator: Roger Bedimo, M.D.

Co-Investigator(s): Henning Drechsler, M.D.; Teresa Moore, PA-C; Diana L. Turner, PA-C; Irfan Farukhi, M.D.

Study Coordinators: Holly Wise and Joyce Ghormley

• Feeling tired (fatigue)	• Abdominal pain
• Vomiting	• Uneven distribution of body fat (lipodystrophy acquired)
• Altered taste (dysgeusia)	• Muscle spasms
• Constipation	• Rash
• Loss/Lack of appetite (anorexia)	• Stomach sticking out (abdominal distension)

♦ Some rare serious side effects judged to be related to Raltegravir or other HIV drugs, given along with Raltegravir are listed below. The rare serious side effects occurred in only one patient except for hypersensitivity which occurred in two.

• Problems caused by low amounts of certain blood cells including low white cells (neutropenia) and low red cells (anemia)	• Kidney failure (renal failure)
• Heart attack (Myocardial infarction)	• Irritation of the stomach lining (gastritis)
• Problems with the kidney (toxic nephropathy)	• Inflamed pancreas (pancreatitis)
• Two separate patients had serious hypersensitivity or drug sensitivity reactions. In both cases the reaction was related to other HIV medications.	• Liver problem (hepatitis)
• Changes in blood chemistry (metabolic acidosis), decrease in kidney function (renal failure), and death	

A condition called Immune Reconstitution Syndrome can happen in some patients with advanced HIV infection (AIDS) when combination antiretroviral treatment is started. Signs and symptoms of inflammation from opportunistic infections that a person has or had may occur as the medicines work to control the HIV infection and strengthen the immune system.

Darunavir

Tests with Darunavir have been performed in laboratories, animals and healthy volunteers, as well as previous clinical studies in HIV-1 infected patients.

Any adverse events described here have occurred in less than 2% of people who have taken Darunavir.

The most frequently seen side effects of Darunavir when taken in combination with ritonavir and other HIV medications (seen in more than 10 percent of subjects) were: These side effects of HIV therapy can often be easily managed by your study doctor.

• Nausea	• Skin Rash*
• Headache	• Diarrhea

* Skin rash has also been seen with other HIV drugs. It is usually mild or moderate in severity, and typically resolves within one week. It does not usually cause patients to stop the medication. However, certain cases of moderate and all cases of severe skin rashes will require you to stop your HIV drug regimen and might need additional study visits. Stevens-Johnson Syndrome (a form of severe skin rash) has been reported in patients taking Darunavir. Stevens-Johnson Syndrome is a rare but serious condition that may cause rash and painful blisters on your skin and mucous membranes (eyes, nose, mouth) and can result in death. If your skin should show any unusual qualities during the study (mainly skin rash, but including unusual boils or blisters) you should inform the study doctor immediately. You should go to the study site for a prompt medical evaluation. It is highly recommended that you see the study doctor as soon as any rash or skin condition appears.

Sulfa Allergy

Darunavir is a type of sulfa drug (sulfonamide). You should tell your study doctor if you have a sulfa allergy since it is recommended to use Darunavir with caution in patients who have sulfa allergies. Your study doctor will talk to you about potential risks of taking this medication when you have a sulfa allergy.

In HIV-1 infected patients, the most common side effects that have been reported while taking antiretrovirals such as, Ritonavir, Tenofovir, or Emtricitabine, are:

- diarrhea
- rash
- nausea
- headache
- feeling tired (fatigue)
- dizziness
- trouble sleeping (insomnia)
- heart attack (myocardial infarction - rare but serious side effect associated with Raltegravir; occurred in one patient during study phase).

Most of these side effects were mild to moderate in nature and went away on their own without treatment.

You will be monitored closely during this trial to detect any possible side effects of the study drug. It is very important that you tell your trial doctor if you experience any side effects while in this study. You will be told as soon as possible of any important new information that may make you change your mind about staying in the research study.

Radiation Exposure

Full Body DXA Scans

DXA scans are equal to about 5 mrem, which means this procedure(s) will expose you to about 5 days of natural background radiation exposure. No increased risk has been scientifically demonstrated from this level of exposure, though a very small increase in cancer risk may exist.

Blood Draw Risks

Blood drawing may cause some discomfort, bleeding or bruising where the needle enters the body. A small blood clot may form where the needle enters the body or there may be swelling in the area. Rarely, fainting or local infection may occur. Care will be taken to prevent these complications.

Unforeseen risks: A previously unknown problem could result from your taking part in this research. There could be an interaction between the study drug and other medications you take (prescribed or over-the-counter). It is not possible to estimate the chances of such problems or how serious the problems could be. Any new findings will be given to you that may affect your willingness to take part in this study. If new findings are discovered, you will be asked to sign a new (updated) informed consent form to document that new information provided in the updated Consent Form has been explained to you.

4. WILL THE RESEARCH BENEFIT ME OR OTHERS?

You may or may not benefit from participating in this research study.

5. WHAT ARE MY ALTERNATIVES TO BEING A RESEARCH SUBJECT?

Alternative treatments or other investigational drugs for your illness are available.

6. WILL I GET PAID?

You will not be paid for your participation in this study.

7. WILL I HAVE TO PAY?

Subjects do not pay for treatment associated with participation in a VA research program.

There is no promise that the drugs you take during this study will be continued after the study is finished. If you are a veteran and can receive care at the VA you may be able to receive the same drug after the study only if the Dallas VAMC has the drug and your physician decides that it is the best treatment for you.

8. DOES BEING PREGNANT OR THE POSSIBILITY OF BEING PREGNANT PREVENT ME FROM TAKING PART?

Every effort will be made to have females enter this study on an equal basis with male subjects. Medically accepted birth control is needed to enter this study. This includes, but is not limited to: abstinence, birth control pills, IUD's, condoms, diaphragms, implants, being surgically sterile, or being in a post-menopausal state. However, no birth control method completely prevents pregnancy. If you become pregnant there may be a high risk of miscarriage, birth defects or other problem for the fetus. If you are female and of child bearing age, you must have a negative pregnancy test before starting the study.

9. WHAT IF I GET INJURED?

The VA has the authority to provide medical treatment to participants injured by participation in a VA study. If you are injured as a result of being in this study, the VA will provide the necessary medical treatment in accordance with federal law. If you want to make a legal claim against the VA or anyone who works for the VA, special laws may apply. The Federal Tort Claims Act (28 U.S.C. 1346(b), 2671-2680) is a federal law that controls when and how a person can bring a claim against the U.S. Government. If you sign this document you are not giving up your right to make a legal claim against the United States.

10. ARE MY RESEARCH RECORDS SAFE FROM THE PUBLIC?

The study doctors keep your research records private in the same way as your other medical records. No one has access to your records except as required by law. You are, however, authorizing Merck & Co., Inc., Tibotec Therapeutics, the Dallas VA Institutional Review Board (IRB), the Dallas VA Research and Development Committee and the members of the Dallas VA

Research Office to inspect your medical and research records. These committees, people, and offices at the Dallas VAMC are responsible for overseeing human research studies.

By signing this form, you will allow the Veterans Health Administration (VHA) to provide Roger Bedimo, M.D. and his research team access to the following health data about you: HIV status, laboratory results, medications you are taking, and past and current medical history.

If you do not sign this form, you will not be part of the study. This approval to use your health data has no expiration date.

VHA complies with the requirements of the Health Insurance Portability and Accountability Act of 1996 and all other laws that protect your privacy. We will protect your health data according to these laws. Despite these protections, there is a possibility that your health data could be used or disclosed in a way that it will no longer be protected. Our Notice of Privacy Practices (a separate document) provides more information on how we protect your health data. If you do not have a copy of the Notice, the research team will provide one to you.

If you choose to take part in the study, certain government agencies (such as the FDA or VA) may look at your research records. Your name as a subject in this study is private, and will not be included in any report prepared as a result of this study.

11. DO I HAVE TO TAKE PART IN THIS STUDY, OR CAN I WITHDRAW FROM THE STUDY?

Taking part in this study is voluntary and you may refuse to take part without penalty or loss of benefits to which you are otherwise entitled. You are free to withdraw your consent and stop taking part at any time. Not taking part in the study will in no way affect the quality of care you receive now or in the future from the VA. This will also not affect your right to take part in other studies. The study doctors will answer any questions you may have about the study.

You can also take back your authorization for the VHA or the study doctors to access or to share your health data with outside parties at any time. To stop taking part in the study or to take back your authorization, you should contact both:

1) Dr. Roger Bedimo or his representative listed at the bottom of this form, and

Subject Name: ______________________________ Date: ______________

Title of Study: Evaluation of Safety & Efficacy of Raltegravir/Darunavir Combination in Antiretroviral-Naïve Patients, #07-124

Principal Investigator: Roger Bedimo, M.D.

Co-Investigator(s): Henning Drechsler, M.D.; Teresa Moore, PA-C; Diana L. Turner, PA-C; Irfan Farukhi, M.D.

Study Coordinators: Holly Wise and Joyce Ghormley

2) the IRB Administrator of the Dallas VA Medical Center [telephone: 214-857-0291]; mail:
Dallas VA Medical Center, IRB Administrator (151)
4500 S. Lancaster Rd.
Dallas, TX 75216.

If you decide to take back your authorization, you will be given a form to show your desire in writing. If you take back your authorization, you will not be able to continue to take part in the study. This will not affect your rights as a VHA patient.

If you take back your authorization, Dr. Bedimo and his research team can keep using health data about you that has been collected. No health data will be collected after you take back the authorization.

Your doctor may also take you out of the study without your consent for medical or administrative reasons. Any significant new findings that develop during the course of the research study that the study doctor thinks may affect your willingness to continue to take part will be given to you as soon as possible.

12. WHOM SHOULD I CONTACT FOR QUESTIONS OR PROBLEMS?

If you have any questions about this study or have any bad effects of your treatment, you should call the study doctor, whose name and contact number appear on the last page of this form.

If you have any questions about whether this is a VA North Texas Healthcare System-approved research study, you may contact the Research Compliance Officer at 214-857-0341.

If you have any questions about your rights as a patient, complaints about your treatment or general concerns about the conduct of the research study, you may contact the Dallas VAMC Patient Representatives at 214-857-0482. The Patient Representative will guide you in resolving your question or complaint.

If you have a medical emergency you should immediately call 911 for assistance.

Subject Name: ______________________ Date: __________

Title of Study: Evaluation of Safety & Efficacy of Raltegravir/Darunavir Combination in Antiretroviral-Naïve Patients, #07-124

Principal Investigator: Roger Bedimo, M.D.

Co-Investigator(s): Henning Drechsler, M.D.; Teresa Moore, PA-C; Diana L. Turner, PA-C; Irfan Farukhi, M.D.

Study Coordinators: Holly Wise and Joyce Ghormley

RESEARCH SUBJECT'S RIGHTS:

I have read or have had read to me all of the above. The study has been explained to me and all of my questions have been answered. If I have questions later, I have been informed that I can contact Dr. Roger Bedimo. I have been told of the risks or discomforts and possible benefits of the study. I have been told of other choices of treatment open to me.

I have been informed that I do not have to take part in this study and my refusal to take part will involve no penalty or loss of rights to which I am entitled. I may withdraw at any time without penalty or loss of VA or other benefits to which I am entitled. The study doctor can take me out of the study at any time if it appears to be medically harmful to me, if I fail to follow directions for taking part in this study, if it is discovered that I do not meet the study requirements, or if the study is canceled.

In case there are medical problems or questions, I have been told I can call Dr. Roger Bedimo at 214-857-0410 or Joyce Ghormley at 214-857-1606 during the day or at 800-725-4436 after hours.

I was informed of my rights as a research subject, and I voluntarily consent to take part in this study. I authorize the use of my identifiable patient health information as described in this form. I will receive a signed copy of this consent form.

Subject's Signature **Date**

Signature of Witness **Date**

Signature of Person Obtaining Informed Consent **Date**

Research Subject's Bill of Rights

1. Be informed of the nature and purpose of the research.

2. Be clearly told of the procedures to be followed in the medical research, and any drug or device to be used.

3. Be clearly told of any discomforts and risks that might be expected from the research.

4. Be clearly told of any benefits that the patient might expect from the research.

5. Be clearly told of any other appropriate procedures, drugs, or devices that might be helpful to the patient, and their risks and benefits.

6. Be clearly told how to get medical treatment, if needed, after the research is finished if problems should arise.

7. Be given the chance to ask any questions about the research or the procedures involved.

8. Be clearly told that consent to take part in the medical research and/or release of identifiable patient health information may be taken back at any time. The patient may stop taking part in the medical research without any penalty or loss of VA or other benefits.

9. Be given a copy of the signed and dated written consent form.

10. Be given the chance to decide to consent or not to consent to a medical research study without any force, fraud, deceit, duress, coercion, or undue influence on the patient's decision.
