# Supplementary material for: The RADAR Study: Week 48 Safety and Efficacy of RAltegravir Combined with Boosted DARunavir Compared to Tenofovir/Emtricitabine Combined with Boosted Darunavir in Antiretroviral-Naive Patients. Impact on Bone Health
Source: PLoS One. 2014 Aug 29;9(8):e106221. doi: 10.1371/journal.pone.0106221 (PMC4149560; doi:10.1371/journal.pone.0106221)
Supplement: Protocol S1 — RADAR study protocol (abbreviated) – Initial. (PDF) [file pone.0106221.s007.pdf]

1. Title of Study: Evaluation of Safety & Efficacy of Raltegravir/Darunavir Combination in Antiretroviral-Naïve Patients

2. Principal Investigator: **Roger Bedimo, M.D.**
Co-Investigators: Henning Drechsler, M.D.
Teresa Moore, PA-C
Diana L. Turner, PA-C

3. Sponsor of the Study: Merck & Co, Inc.

4. Investigational New Drug (IND)/Investigational Device Exemption (IDE): This study uses all currently licensed HIV medications.

5. Purpose of the Study, Including the Hypothesis to be Tested: Raltegravir is a leading candidate in a new class of antiretroviral medications called integrase inhibitors. Inhibition of integrase prevents the HIV DNA virus from entering into the human DNA genome, thus blocking the ability of HIV to reproduce and spread in the body. It is taken orally twice a day (every twelve hours) and does not require the addition of low-dose Ritonavir to achieve maximum beneficial effect.

Raltegravir has been shown to have excellent virologic effectiveness in patients who have not yet taken antiretrovirals (treatment naïve) and in heavily treatment experienced patients. It has also been shown to have unusually rapid virologic response. This result might be excellent in delaying viral resistance in naïve patients.

6. Background and Results of Previous Related Research: HAART regimens with the most experience in demonstrating virologic and immunologic efficacy – and therefore recommended by the DHHS for initial therapy of antiretroviral naïve patients – are those composed of 2 NRTI and 1 NNRTI a PI (with or without ritonavir boosting). However, because of concerns about long-term drug toxicity, the development of drug resistance, and potential complications in pregnant women, it is imperative that other drug combinations, including NRTI-free regimens, be investigated as possible alternative initial regimens.

Possessing a novel mechanism of action, Raltegravir has been found to have high efficacy and tolerability in combination with two nucleoside analogues in naïve patients, and with optimized background in highly experienced patients. The combination of Raltegravir with a potent PI with a mild metabolic profile will likely yield an even better response among naïve patients, while preventing treatment-limiting toxicities.

7. Definition of the Population to Which the Study is Directed, with Justification: Treatment naive HIV-1 infected subjects who are ready to begin antiretroviral medication.

8. Subject Selection, Inclusion/Exclusion Criteria:

Inclusion Criteria:

- The patient has documented HIV-1 infection.
- The patient is at least 18 years of age.
- Antiretroviral naive, defined as 7 days or less of ARV treatment at any time prior to study entry.
- HIV viral load greater than 5,000 copies/ml within 90 days of study entry
- CD4 count is greater than 100/ml within 90 days of study entry
- Willing to use acceptable forms of contraception
- Parent or guardian willing to provide informed consent, if applicable
- Hepatitis B surface antigen (HBsAg) negative at study entry

Exclusion Criteria:

- Patient is current participant in a Raltegravir trial or in trials involving any of the other study medications (Darunavir, Tenofovir or Emtricitabine).
- Immunomodulators (e.g., interleukins, interferons, cyclosporine), HIV vaccine, systemic cytotoxic chemotherapy, or investigational therapy within 30 days prior to study entry. Individuals receiving either stable physiologic glucocorticoid doses, corticosteroids for acute therapy for pneumocystis pneumonia, or a short course (2 weeks or less) of pharmacologic glucocorticoid therapy will not be excluded.
- Known allergy/sensitivity to study drugs or their formulations
- Patient has a condition (including but not limited to active alcohol or drug use) that, in the opinion of the investigator, may interfere with patient adherence or safety
- Patient with acute hepatitis due to any cause or clinically significant chronic liver disease including but not limited to cirrhosis, ascites, encephalopathy, hypoalbuminemia, prolonged PT/PTT and/or esophageal varices.
- Patient has severe renal insufficiency defined as a calculated creatinine clearance at time of screening <30 mL/min, base on Cockcroft/Gault equation which is as follows (and 0.85 X this value for females):
    - CrCl (mL/min) = [(140-Age) x Weight (in Kg)]/72 x Serum Creatinine (mg/mL)
- Serious illness requiring systemic treatment or hospitalization. Patients who have completed therapy or are clinically stable on therapy for at least 7 days prior to study entry are not excluded.
- Known clinically relevant cardiac conduction system disease

- Patient requires or is anticipated to require any of the prohibited medications noted in the protocol

- Current imprisonment or involuntary incarceration for psychiatric or physical (e.g., infectious disease) illness

Pregnancy and Breastfeeding. Women who become pregnant during the study will be required to permanently discontinue their study regimens.

9. Number of Subjects in the Study: 80 patients will be enrolled at this site.

10. Justification for Use of Special Subject Populations: N.A.

11. Study Design: This is a randomized, active Control, safety/efficacy study. All eligible patients (antiretroviral naïve,) will be randomized (1:1) into two treatment groups:

1. Group A: will receive Raltegravir + Ritonavir-boosted Darunavir
2. Group B: will receive Tenofovir + Emtricitabine + Ritonavir-boosted Darunavir

12. Description of Procedures to be Performed: The investigator will follow the patients according to the standard of care for HIV infection. Laboratory assessments (including HIV RNA and CD4 cell count) will be performed locally at each study visit and at the investigator's discretion thereafter. A chest x-ray may be performed at screening if the patient's last chest x-ray is more than 4 weeks prior to screening. At each clinic visit the study team will monitor for side effects, check vital signs, and perform blood tests for safety labs and pregnancy tests for females.

13. Anticipated Data and Data Analysis:

Primary Outcome Measures:

- Time from randomization to virologic failure (HIV viral load of 1,000 copies/ml or greater at or after Week 16 and before Week 24 or 400 copies/ml or greater at or after Week 24.

Secondary Outcome Measures:

- Percentage of patients with occurrence of fasting hypertriglyceridemia (TG >200) or hypercholesterolemia (TC >240) at week 48

- Median change from baseline in components of lipid panel at Weeks 48

- Percentage of patients with serious adverse experience

- Percentage of patients with drug-related adverse experiences that result in grade 3 or above laboratory toxicity

- Percentage of patients with drug related adverse experiences that lead to treatment interruption

- Time from treatment dispensation to the first development of a Grade 3 or 4 sign, symptom, or laboratory abnormality that is at least one grade higher than at baseline

- Percentage of patients with HIV viral load levels less than 50 and less than 400 copies/ml at 24 and 48 weeks.

- Median CD4 count at 24 and 48 weeks and at virologic failure

- HIV-1 drug resistance patterns at baseline and at time of virologic failure

For assessment of safety and tolerability, counts and percentages of patients with clinical or laboratory adverse experiences of the following type will be tabulated (1) serious adverse experience, (2) drug-related adverse experiences that result in grade 3 or above laboratory toxicity; (3) drug related adverse experiences that lead to treatment interruption; (4) drug related adverse experiences leading to discontinuation.

<u>Exploratory analyses:</u>

- Percentage of patients with occurrence of fasting hypertriglyceridemia (TG >200) or hypercholesterolemia (TC >240) at week 48

- Median change from baseline in components of lipid panel at Weeks 48

- Virologic and immunologic response, safety, and tolerability by race/ethnicity, age, gender, and hepatitis B and C coinfection

- Occurrence of targeted clinical events, including death, AIDS-defining illness, and HIV-1 related events (including the CDC Category B diseases)

14. Provisions for Managing Adverse Reactions: The risks to subjects entailed by this study are those minimal risks due to blood drawing for clinical monitoring and those generally accepted due to the administration of licensed antiretrovirals.

The total volume of blood drawn is consistent with clinical standard of care. The volume of blood drawn will be no more than 35 ml or approximately 2-3 tablespoons.

15. Risk/Benefit Assessment: In HIV-1 infected patients, the most common side effects that have been reported while taking antiretrovirals such as the medications used in this protocol are diarrhea, rash, nausea, headache, feeling tired (fatigue), dizziness, trouble sleeping (insomnia). Most of these side effects are mild to moderate in nature and resolve on their own without treatment. All patients will be monitored closely for adverse effects and interventions will be made as necessary. The patients will be instructed on the procedures for reporting adverse events and/or side effects.

Participation in this protocol is considered minimal risk as all medications used in this protocol are licensed for public use. The patients may not benefit directly from participating in this protocol.

Safety data of serious adverse experiences and drug-related adverse experiences that result in Grade 3 or above laboratory toxicity, lead to treatment interruption, or discontinuation will be collected on in-house case report forms. The laboratory safety assessments should include a chemistry evaluation with serum creatinine, liver function tests, complete blood count and a lipid profile. Additional laboratory assessments would be performed per standard of care at the investigator's discretion. In addition, all patients that experience virological failure or who discontinue from the study early should have the 14-day post-therapy follow-up visit noted on the study flow chart (Section 1.6).

16. Process for obtaining informed consent and Protecting Patient Privacy: Informed consent will be obtained by the principal investigator or his designees, who will explain the rationale and details of the study to eligible subjects, and invite their participation. Those consenting will sign an approved informed consent.

17. Documentation of Informed Consent: The P.I., Co-Investigator's, or the Study Coordinator will have equal accessibility to approach a potential subject for this research study. The Study Coordinator, along with the P.I., will obtain written consent and the Study Coordinator will document this process in CPRS following the PPHRS guidelines. Subjects will not be considered for participation in this protocol if they have a diminished mental capacity. If the subject is not English-speaking a translator will be provided prior to obtaining written consent.

18. Payment to Subjects for Their Participation: The study participants will not be compensated for their participation in this protocol.

19. Provisions for Data Storage and Confidentiality: Participation will be completely confidential. Information gained in this study will be shared with other researchers and sponsors (e.g. drug companies, the Food and Drug Administration), or presented at meetings or in publications, but patient identity will not be revealed in any way. All patient related documents will be kept in locked files in the Study Coordinator's office. Only the P.I., Co-Investigator's and the Study Coordinator will have access to these files. The subject's will be identified by a random number, which will not be linked to the subject's confidential information.

In accordance with HIPAA, the consent form describes to the subject what protected health information (PHI) will be obtained and/or stored and for what purpose, as well as a list of who may have access to this data, including outside agencies. All records will be maintained in a locked cabinet in the research team's locked office. Computerized data will be de-identified and stored on a password protected computer in a locked office of the research team.

In accordance with VA guidelines, all records of this research study will continue to be securely maintained for a minimum of five years from the date of completion of the study. The records will be kept in a locked file cabinet or locked room with limited access. If the PI leaves the VA facility, the original research records will be retained by the institution.

If the sponsor requires longer retention of the research records, the records will only be returned to the sponsor after the required minimum retention time of VA has been met. At that time, research records will be returned to the sponsor or destroyed upon approval of the sponsor.

20. Dissemination of Research Results: The results from this research will be published in medical journals and presented at appropriate meetings.

IRB Approved
1/07/08-01/06/09
