# Supplementary material for: The RADAR Study: Week 48 Safety and Efficacy of RAltegravir Combined with Boosted DARunavir Compared to Tenofovir/Emtricitabine Combined with Boosted Darunavir in Antiretroviral-Naive Patients. Impact on Bone Health
Source: PLoS One. 2014 Aug 29;9(8):e106221. doi: 10.1371/journal.pone.0106221 (PMC4149560; doi:10.1371/journal.pone.0106221)
Supplement: Protocol S2 — RADAR study protocol – Revised. (PDF) [file pone.0106221.s008.pdf]

1. SUMMARY

1.1. TITLE

Safety and Efficacy of Raltegravir + Darunavir/Ritonavir vs. Emtricitabine/Tenofovir + Darunavir/Ritonavir for Antiretroviral-Naïve Patients.

1.2. INDICATION

Raltegravir has recently been approved for the treatment of HIV-1 infection in treatment-experienced patients

1.3. SUMMARY OF RATIONALE

HAART regimens with the most experience in demonstrating virologic and immunologic efficacy – and therefore recommended by the DHHS for initial therapy of antiretroviral naïve patients – are those composed of 2 NRTI and 1 NNRTI or PI with ritonavir boosting. However, because of concerns about long-term drug toxicity, the development of drug resistance, and potential complications in pregnant women, it is imperative that other drug combinations, including NRTI-free regimens, be investigated as possible alternative initial regimens.

Possessing a novel mechanism of action, Raltegravir has been found to have high efficacy and tolerability in combination with two nucleoside analogues in naïve patients, and with optimized background in highly experienced patients. Darunavir is the newest PI which has recently been shown to have excellent efficacy and tolerability in antiretroviral naïve patients. The combination of Raltegravir and a potent PI with an improved metabolic profile will likely yield an even better response among naïve patients, while preventing treatment-limiting toxicities.

1.4. SUMMARY OF STUDY DESIGN

This is a randomized, active control, safety/efficacy pilot estimation study. Eighty patients will be randomized 1:1 into two different treatment groups:

1. Group A: will receive Raltegravir + Ritonavir-boosted Darunavir
2. Group B: will receive Tenofovir + Emtricitabine + Ritonavir-boosted Darunavir

1.5. DOSAGE/DOSAGE FORM, ROUTE, AND DOSE REGIMEN

All study medications will be taken as recommended by manufacturer.

a. Raltegravir: 400 mg twice daily, without regard to food. No recommendation for dosage adjustment in patients with severe renal impairment, mild or moderate hepatic dysfunction.
b. Darunavir: 800 mg once daily plus ritonavir 100 mg once daily; administer with food. No recommendation for dosage adjustment in patients with severe renal impairment or end stage renal disease or severe hepatic dysfunction.
c. Tenofovir: 300 mg once daily. No dosage adjustment required for hepatic impairment. Renal impairment: Cl_{cr} 30-49 mL/minute: 300 mg every 48 hours; Cl_{cr} 10-29 mL/minute: 300 mg twice weekly; Cl_{cr} <10 mL/minute without hemodialysis: No recommendation available; Hemodialysis: 300 mg every 7 days

IRB Approved
06/24/13

or after a total of 12 hours of dialysis (usually once weekly assuming 3 dialysis sessions lasting about 4 hours each)

d. Emtricitabine: 200 mg once daily. No adjustment required for hepatic impairment. Renal impairment: Cl_{cr} 30-49 mL/minute: 200 mg every 48 hours; Cl_{cr} 15-29 mL/minute: 200 mg every 72 hours; Cl_{cr} <15 mL/minute (including hemodialysis patients): 200 mg every 96 hours; administer after dialysis on dialysis days

IRB Approved
06/24/13

1.6. STUDY FLOW CHART

TRIAL PERIOD	SCREENING	TREATMENT									
**Week***	**(-4 to -3)**	**0**	**2**	**4**	**8**	**16**	**24**	**32**	**40**	**48**	**EOT**
**Day**	**(-28)**	**0**	**(14+/-2)**	**(28+/-2)**	**(56+/-4)**	**(112+/-4)**	**(168+/-4)**	**(224+/-4)**	**(280+/-4)**	**(336+/-4)**	
**Visit**	**1**	**2**	**3**	**4**	**5**	**6**	**7**	**8**	**9**	**10**	**11**
Informed Consent	X										
Medical History and Baseline Conditions	X										
Physical Examination	X	X	X	X	X	X	X	X	X	X	X
Weight and BMI Calculation	X	X			X	X	X	X	X	X	X
Eligibility Criteria	X	X									
CBC	X	X	X	X	X	X	X	X	X	X	X
Metabolic Profile	X	X	X	X	X	X	X	X	X	X	X
Viral Load	X	X	X	X	X	X	X	X	X	X	X
CD4+ and CD8+ Cell Counts	X	X	X	X	X	X	X	X	X	X	X
Fasting Blood Glucose	X	X	X	X	X	X	X	X	X	X	X
Fasting Insulin Level	X					X		X		X	X
Fasting Lipid Panel	X	X	X	X	X	X	X	X	X	X	X
Urinalysis	X	X	X	X	X	X	X	X	X	X	X
Lipoprotein Analysis	X					X		X		X	X
LFTs	X	X	X	X	X	X	X	X	X	X	X
Hepatitis Serology	X										
HIV Genotyping	X					X					
HIV Phenotyping	X					X					
Adverse Events	X	X	X	X	X	X	X	X	X	X	X
Obtain Plasma Sample for Storage	X	X	X	X	X	X	X	X	X	X	
Serum Pregnancy Test	X	X	X	X	X	X	X	X	X	X	X
Randomization		X									
Dispense Darunavir/Raltegravir		X		X	X	X	X	X	X	X	
Concomitant Medications	X	X	X	X	X	X	X	X	X	X	X
Adherence Questionnaire and Pill Count				X	X	X	X	X	X	X	
Whole Body DEXA Scan		X								X	

IRB Approved
06/24/13

2. CORE PROTOCOL

2.1 OBJECTIVES AND HYPOTHESES

2.1.1 Hypotheses

We hypothesize that the virologic efficacy (time to loss of virologic response) at 24 weeks will be at least as high following a regimen of Raltegravir + boosted Darunavir as with a regimen of Tenofovir + Emtricitabine + boosted Darunavir.

We further hypothesize that a regimen of Raltegravir + boosted Darunavir will not result in higher rates of adverse events at 24 weeks than a regimen of Tenofovir + Emtricitabine + boosted Darunavir.

2.1.2 Primary Objective

The primary objective is to determine whether a regimen of Raltegravir + Ritonavir-boosted Darunavir achieves a comparable virologic efficacy (TLOVR) at 24 weeks to Tenofovir + Emtricitabine + Ritonavir-boosted Darunavir

2.1.3 Secondary Objectives

a. Determine whether Raltegravir + Ritonavir-boosted Darunavir at 48 weeks is associated with a median change in CD4 count from baseline comparable to Tenofovir + Emtricitabine + boosted Darunavir
b. Compare the lipid profiles of patients receiving Raltegravir + Ritonavir-boosted Darunavir with those receiving Tenofovir + Emtricitabine + boosted Darunavir at week 48
c. Determine changes in lipoprotein particles profile, as defined by their apolipoprotein composition and lipoprotein particle size distribution,(LDL subtypes 1 through 7 and HDL subtypes), in both treatment arm at weeks 16, 32 and 48.
d. Determine any changes from baseline to week 48, in insulin resistance by homeostasis model assessment (HOMA-IR) derived from fasting plasma glucose and insulin levels
e. Determine any changes in percent of body fat, lean body mass, and bone density on whole body DEXA scans between baseline and week 48.

2.2 SUBJECT/PATIENT INCLUSION CRITERIA

Inclusion Criteria:

- The patient has documented HIV-1 infection.
- The patient is at least 18 years of age.
- Antiretroviral naive, defined as 7 days or less of ARV treatment at any time prior to study entry.
- HIV viral load greater than 5,000 copies/ml prior to study entry.
- Willing to use acceptable forms of contraception
- Hepatitis B surface antigen (HBsAg) negative at study entry

2.3 SUBJECT/PATIENT EXCLUSION CRITERIA

IRB Approved
06/24/13

Exclusion Criteria:

- Patient is current participant in a Raltegravir trial or in trials involving any of the other study medications (Darunavir, Tenofovir or Emtricitabine)
- Immunomodulators (e.g., interleukins, interferons, cyclosporine), HIV vaccine, systemic cytotoxic chemotherapy, or investigational therapy within 30 days prior to study entry. Individuals receiving either stable physiologic glucocorticoid doses, corticosteroids for acute therapy for pneumocystis pneumonia, or a short course (2 weeks or less) of pharmacologic glucocorticoid therapy will not be excluded.
- Known allergy/sensitivity to study drugs or their formulations
- Patient has a condition (including but not limited to active alcohol or drug use) that, in the opinion of the investigator, may interfere with patient adherence or safety
- Patient with acute hepatitis due to any cause or clinically significant chronic liver disease (defined as Child-Pugh Class B or C; see table 3)
- Patient has severe renal insufficiency defined as a calculated creatinine clearance at time of screening <30 mL/min, base on Cockcroft/Gault equation which is as follows (and 0.85 X this value for females):
    - CrCl (mL/min) = [(140-Age) x Weight (in Kg)]/72 x Serum Creatinine (mg/mL)
- Serious illness requiring systemic treatment or hospitalization. Patients who have completed therapy or are clinically stable on therapy for at least 7 days prior to study entry are not excluded.
- Known clinically relevant cardiac conduction system disease
- Patient requires or is anticipated to require any of the prohibited medications noted in the protocol
- Current imprisonment or involuntary incarceration for psychiatric or physical (e.g., infectious disease) illness
- Pregnancy and Breastfeeding. Women who become pregnant during the study will be required to permanently discontinue their study regimens.

2.4 STUDY DESIGN AND DURATION

This is a randomized, active Control, safety/efficacy study. All eligible patients (antiretroviral naïve,) will be randomized (1:1) into two treatment groups:

1. Group A: will receive Raltegravir + Ritonavir-boosted Darunavir
2. Group B: will receive Tenofovir + Emtricitabine + Ritonavir-boosted Darunavir

For analysis, patients will also be stratified according to baseline viral load (VL <100,000 or ≥100,000) and baseline CD4 count (<200 or ≥200).

Study Endpoints:

Primary Outcome Measures:

IRB Approved
06/24/13

- Time from randomization to virologic failure (HIV viral load of 1,000 copies/ml or greater at or after Week 16 and before Week 24, or two consecutive HIV viral load of 50 copies/ml or greater at or after Week 24)

Secondary Outcome Measures:

- Percentage of patients with serious adverse experience
- Percentage of patients with drug-related adverse experiences that result in grade 3 or above laboratory toxicity
- Percentage of patients with drug related adverse experiences that lead to treatment interruption
- Time from treatment dispensation to the first development of a Grade 3 or 4 sign, symptom, or laboratory abnormality that is at least one grade higher than at baseline
- Percentage of patients with HIV viral load levels less than 50 copies/ml at 24 and 48 weeks.
- Median CD4 count at 24 and 48 weeks and at virologic failure
- Median change in limb fat by DEXA at week 48
- Percentage of patients with occurrence of fasting hypertriglyceridemia (TG >400) or hypercholesterolemia (TC >240) at week 48
- HIV-1 drug resistance patterns at baseline and at time of virologic failure

For assessment of safety and tolerability, counts and percentages of patients with clinical or laboratory adverse experiences of the following type will be tabulated (1) serious adverse experience, (2) drug-related adverse experiences that result in grade 3 or above laboratory toxicity; (3) drug related adverse experiences that lead to treatment interruption; (4) drug related adverse experiences leading to discontinuation.

Exploratory analyses

- Median change from baseline in components of lipid panel at Weeks 48
- Changes in glucose tolerance as measured by the HOMA-IR
- Changes in lipoprotein profile and LDL and HDL particle size and distribution
- Virologic and immunologic response, safety, and tolerability by race/ethnicity, age, gender, and hepatitis C coinfection
- Occurrence of targeted clinical events, including death, AIDS-defining illness, and HIV-1 related events (including the CDC Category B diseases)

2.4.1 Summary of Study Duration

The study will last until fully enrolled (80 patients). The expected duration is about 12 months.

2.4.2 Treatment Plan

Eighty number patients will be randomized 1:1 to one of the two study regimens. The treatment allocations will be unblinded/open-label. The patients will take study medications in the following doses :

IRB Approved
06/24/13

a. Raltegravir 400 mg twice a day, without regard to food consumption.
b. Darunavir: 800 mg once daily plus ritonavir 100 mg once daily; administer with food. No recommendation for dosage adjustment in patients with renal or severe hepatic dysfunction.
c. Tenofovir: 300 mg once daily. No dosage adjustment required for hepatic impairment. Renal impairment: Cl_{cr} 30-49 mL/minute: 300 mg every 48 hours; Cl_{cr} 10-29 mL/minute: 300 mg twice weekly; Cl_{cr} <10 mL/minute without hemodialysis: No recommendation available; Hemodialysis: 300 mg every 7 days or after a total of 12 hours of dialysis (usually once weekly assuming 3 dialysis sessions lasting about 4 hours each)
d. Emtricitabine: 200 mg once daily. No adjustment required for hepatic impairment. Renal impairment: Cl_{cr} 30-49 mL/minute: 200 mg every 48 hours; Cl_{cr} 15-29 mL/minute: 200 mg every 72 hours; Cl_{cr} <15 mL/minute (including hemodialysis patients): 200 mg every 96 hours; administer after dialysis on dialysis days

The investigators will follow the patients according the standard of care. In addition, the patients will be followed according the above flow chart: Study visits will be performed at screening, enrollment (week 0) and weeks 2, 4, 8, 16, 24, 32, 40 and 48.

2.5 LIST OF SAFETY MEASUREMENTS

Safety data of serious adverse experiences and drug-related adverse experiences that result in Grade 3 or above laboratory toxicity, lead to treatment interruption, or discontinuation will be collected on case report forms. The laboratory safety assessments should include a chemistry evaluation with serum creatinine, liver function tests, complete blood count and a lipid profile. Additional laboratory assessments would be performed per standard of care at the investigator’s discretion. In addition, all patients that experience virological failure or who discontinue from the study early should have the 14-day post-therapy follow-up visit noted on the study flow chart (Section 1.6)

2.6 DATA ANALYSIS SUMMARY

The primary analysis of efficacy and safety will be based upon the All Patients As Treated (APaT) approach, which includes all patients who received one or more doses of the test drug therapy. All patients who take study medication will be included in the analysis of safety and tolerability. Only those adverse experiences that occur while on study therapy or within 14 days after discontinuation of study therapy will be included in the analysis.

This is an estimation study. The primary endpoint is the comparison of the efficacy of the test regimen (Raltegravir + Darunavir) to Tenofovir + Emtricitabine + Darunavir. The mean effects of the test regimen (Raltegravir + Darunavir) will be compared to comparator regimen.

The analysis will be time to loss of virologic response (TLOVR) [12]. This analysis defines responders as subjects maintaining a minimum of two sequential HIV viral load <50 through the scheduled visits without intervening replicated rebounds or treatment discontinuations.

IRB Approved
06/24/13

Logistic regression analysis will be used to analyze outcome data to determine the difference between the two regimens. With 40 patients enrolled in each arm, the study has 80% power to rule out a 15% difference in efficacy between the two regimens. This assumes a true response rate of 85% for both treatment groups.

For assessment of safety and tolerability, counts and percentages of patients with clinical or laboratory adverse experiences of the following type will be tabulated: (1) serious adverse experience; (2) drug-related adverse experiences that result in grade 3 or above laboratory toxicity; (3) drug related adverse experiences that lead to treatment interruption; (4) drug related adverse experiences leading to discontinuation.

3. PROTOCOL DETAILS

3.1 RATIONALE

HAART regimens with the most experience in demonstrating virologic and immunologic efficacy – and therefore recommended by the DHHS for initial therapy of antiretroviral naïve patients – are those composed of 2 NRTI and 1 NNRTI or a boosted PI . Apart from limited data on PI monotherapy [1, 2], and switch studies [3], NRTI-free regimens have never been used on a significant number of patients for a significant amount of time. Such regimens have usually been found to have significantly lower virologic efficacy [1] However, because of concerns about long-term drug toxicity, the development of drug resistance, and potential complications in pregnant women, it is imperative that other drug combinations, including NRTI-free regimens, be investigated as possible alternative initial regimens.

NRTI use is associated with significant toxicity, including mitochondrial dysfunction (mostly attributed to thymidine-analogue NRTIs): lipoatrophy, peripheral neuropathy, pancreatitis, and lactic acidosis [4]. There's also the risk of hypersensitivity reaction from Abacavir, and caution is needed when using Tenofovir in patients with renal failure. Recent data (including ACTG trials 5110, 5125s, and 5142) suggest that a switch to NRTI-free regimens might significantly improve the side-effect profile [3, 5]. NRTI-free arms had lower rates of lipoatrophy, but also higher rates of hypertriglyceridemia and a trend towards higher virologic failure rate [5]. The NRTI-free regimens in all these studies were Lopinavir with an NNRTI (Efavirenz or Nevirapine), and it's reasonable to expect that the use of a PI with a better metabolic profile than LPV/r might significantly improve the side-effect profile of such regimens.

A suggestion from these and other trial results is that NRTIs might be associated with other than the known complications, but these have been difficult to detect because there have been few long-term comparisons of NRTI-containing and NRTI-free regimens, and very few have been done on naïve HIV-infected patients [6]. Finding effective NRTI-free regimens would have a number of potential benefits including: 1) a significant expansion of therapeutic options; despite the growing number of antiretrovirals, treatment options might still be significantly limited in a patient with a number of baseline NRTI mutations [7] or poor NRTI tolerance; 2) potential avoidance of toxicities;

Raltegravir is a leading candidate in a new class of antiretroviral medications called integrase inhibitors. Inhibition of integrase prevents insertion of HIV DNA into the human

IRB Approved
06/24/13

DNA genome, thus blocking the ability of HIV to replicate. It is administered orally every twelve hours and does not require boosting with low-dose ritonavir to achieve therapeutic concentrations. Raltegravir is not a potent inhibitor or inducer of cytochrome P (CYP) 3A4, and it is predominantly metabolized by glucuronidation, specifically by the enzyme UDP-glucuronosyltransferase (UGT) 1A1.

Raltegravir has been shown to have excellent virologic efficacy in naïve and heavily treatment experienced patients. It also has been shown to have unusually rapid virologic response [8]. This profile might be excellent in delaying emergence of viral resistance in naïve patients.

A two-part, Phase II, dose-ranging trial in treatment-naive patients compared 10-day Raltegravir monotherapy in 28 patients with placebo in seven patients.

Thirty-five patients were enrolled (6–8 patients per treatment group) and completed 10 days of therapy; the mean baseline log10 HIV RNA level ranged from 4.5 to 5.0 copies/mL in each group. On day 10, the mean decrease from baseline in the log10 HIV RNA level was 20.2 copies/mL for the placebo group and 21.9, 22.0, 21.7 and 22.2 log10 copies/mL for the MK-0518 100-, 200-, 400-, and 600-mg treatment groups, respectively. All dose groups had superior antiretroviral activity compared with placebo (P , 0.001 for comparison of each dose with placebo). At least 50% of patients in each MK-0518 dose group achieved an HIV RNA level ,400 [8, 9]. In the second part of the study, 198 treatment-naïve patients (including the initial 30 patients) were randomly assigned to receive either the same twice-daily dosages of Raltegravir or efavirenz 600 mg once daily, both in combination with tenofovir and lamivudine. After 24 weeks of therapy, 85% to 95% of patients on Raltegravir-based regimens achieved viral loads of less than 50 copies/ml across all dosages. In the efavirenz-based regimen, 92% of patients achieved viral loads less than 50 copies/ml. However, viral load reduction was achieved more quickly with Raltegravir regimens.[7]

Three phase III trials of Raltegravir in treatment experienced patients have been conducted (BENCHMRK trials).[10, 11] In both of these studies, more than 75 percent of patients receiving Raltegravir plus optimized background therapy (OBT) achieved viral load (HIV RNA) reductions to less than 400 copies/mL compared to more than 40 percent of patients receiving placebo plus OBT (BENCHMRK-1, 77 percent of patients (N=232) receiving Raltegravir plus OBT vs. 41 percent of patients (N=118) receiving placebo plus OBT; and BENCHMRK-2, 77 percent of patients (N=230) receiving Raltegravir plus OBT vs. 43 percent of patients (N=119) receiving placebo plus OBT, p<0.001 for both studies respectively). Both studies also showed that after 16 weeks of treatment, Raltegravir plus OBT was generally well tolerated. In addition, there were few discontinuations due to adverse experiences (BENCHMRK-1, four patients receiving Raltegravir plus OBT and four patients receiving placebo plus OBT; for BENCHMRK-2, five patients receiving Raltegravir plus OBT and one patient receiving placebo plus OBT).

In subgroup analysis of the BENCHMRK trials, use of Raltegravir and Darunavir was associated with 90% virologic responses (HIV RNA < 400 copies/mL) at 24 weeks in treatment experienced subjects.

Raltegravir is primarily metabolized by the liver via glucuronidation and is neither an inducer nor an inhibitor of CYP3A4. This should clearly reduce potential drug

IRB Approved
06/24/13

interactions with other commonly used antiretroviral agents, especially the currently available protease inhibitors and non-nucleoside reverse transcriptase inhibitors. Specifically, there is no potential or need for boosting with CYP3A4 inhibitors such as ritonavir. Also, unlike most protease inhibitors, and even NNRTIs, Raltegravir does not seem to increase lipid levels.
In protocol 004, Raltegravir (administered in doses ranging from 100 mg bid to 600 mg bid), in combination with Tenofovir and Emtricitabine for 24 weeks was not associated with any elevation in total cholesterol, LDL-cholesterol or triglyceride levels, in contrast to Efavirenz in combination with Tenofovir and Emtricitabine.[10, 11] (Teppler et al., 46th ICAAC, poster 0256a). The combination of Raltegravir with a potent PI with a mild metabolic profile will likely yield an even better response among naïve patients, while preventing treatment-limiting toxicities. This is likely to be the first real chance at a potent NRTI-free regimen for use in naïve patients.

Darunavir is currently approved for use in HAART-experienced patients at the dose of 600 mg bid with ritonavir boosting.
Darunavir is the newest PI approved for patients who are highly treatment-experienced or have HIV-1 strains resistant to multiple PIs based on demonstrated activity against PI-resistant viruses.
The safety and efficacy of Darunavir has been extensively explored, primarily in the POWER 1, 2 and 3 trials (Darunavir/ritonavir compared to other PIs in treatment-experienced patients), the TITAN trial (Darunavir/ritonavir compared to Lopinavir/ritonavir in treatment-experienced patients) and the ARTEMIS trial (Darunavir/ritonavir compared to lopinavir/ritonavir in treatment-naïve patients).

POWER 1 and 2 are two randomised, multinational, phase IIB studies conducted to evaluate the efficacy and safety of darunavir in combination with low-dose ritonavir in treatment-experienced HIV-1-infected patients. A pooled subgroup analysis of their results at week 48 for patients receiving the recommended dose of darunavir-ritonavir compared with those receiving other protease inhibitors (PIs) was recently published [13].

At week 48, 67 of 110 (61%) darunavir-ritonavir patients compared with 18 of 120 (15%) of control PI patients had viral load reductions of 1 log10 copies per mL or greater from baseline (primary endpoint; difference in response rates 46%, 95% CI 35%-57%, p<0·0001). Based on a logistic regression model including stratification factors (baseline number of primary PI mutations, use of enfuvirtide, baseline viral load) and study as covariates, the difference in response was 50% (odds ratio 11·72, 95% CI 5·75-23·89). In the darunavir-ritonavir group, rates of adverse events were mostly lower than or similar to those in the control group when corrected for treatment exposure. No unexpected safety concerns were identified.

In the TITAN trial, 595 treatment-experience LPV/r and DRV/r-naïve patients were randomized to receive DRV/r (600 mg/100 mg bid) vs LPV/r (400 mg/100 mg bid) + optimized background. After 48 weeks a significantly higher proportion of people assigned to darunavir had a viral load below 400 (77% vs. 67%) or 50 copies (71% vs. 60%). Darunavir was found to be non-inferior and superior to lopinavir (P = 0.008).

The ARTEMIS study compared darunavir/ritonavir (800/100mg once daily) with lopinavir/ritonavir (once or twice daily), both in combination with tenofovir/emtricitabine,

IRB Approved
06/24/13

in a randomized, open-label, non-inferiority trial [14]. The study enrolled 689 treatment-naive subjects with a median CD4 count of 228 cells/mm^3, and a median plasma HIV RNA level of 60,000-70,000 copies/mL. At 48 weeks, plasma HIV RNA was <50 copies/mL in 84% of darunavir/ritonavir recipients and 78% of lopinavir/ritonavir recipients (p <0.001). In subgroup analysis, DRV/r QD was superior to LPV/r (overall) in patients with baseline viral load ≥100,000 copies/mL. The DRV/r QD group experienced a lower incidence of lipid abnormalities than the lopinavir/ritonavir group. Grade 2 to 4 adverse events, primarily diarrhea, were seen in 7% of darunavir/ritonavir recipients and 14% of lopinavir/ritonavir recipients (p ,0.01).
Moreover, trough concentrations of once-daily darunavir/ritonavir stayed above the 50% effective concentration (EC50) for wild-type (nonmutant) virus in everyone taking these protease inhibitors (PIs) in the ARTEMIS trial of first-line regimens [15]

We therefore plan to analyze the safety and efficacy of Raltegravir and once-daily boosted Darunavir in HAART-naïve patients, compared to an approved DHHS first-line regimen.

3.2 STUDY PROCEDURES

3.2.1 Concomitant Medication(s)/Treatment(s)

The study patients cannot be concomitantly on any other antiretroviral drugs than the ones outlined in the respective treatment arms. The concomitant use of other medications/therapies is allowed unless specifically prohibited in the Prohibited Concomitant Medications/Therapies section below.

Prohibited Concomitant Medications/Therapies

Raltegravir is eliminated mainly via the UDP-glucuronosyltransferase (UGT)1A1-mediated glucuronidation pathway and, therefore, the compound may be subject to drug-drug interactions when co-administered with drugs that are known to be UGT1A1 inducers or inhibitors. However, Raltegravir is not anticipated to affect the metabolic clearance of drugs metabolized by UGT1A1 given its low UGT1A1 inhibitory (IC50 for the inhibition of UGT1A1 >50 μM) and induction potential. Since Raltegravir is neither an inducer nor inhibitor of cytochrome P-450 enzymes, it is not expected to result in metabolic drug interactions with substrates of cytochromes P-450.

1. The medications/therapies below are contraindicated in this study because they are potent broad inducers of drug metabolism, inducers of CYP3A (thus potential inducers of glucuronidation), and their coadministration with Raltegravir will likely result in altered (lowered) drug levels of Raltegravir:
    a. Phenobarbital
    b. Phenytoin
    c. Rifampin
2. Medications/therapies that may adversely affect the drug levels of the other ARTs used in the three study arms should be avoided to ensure adequate therapeutic levels of all ARTs in the combinations. Medications specifically contraindicated with concomitant use of these other ARTs are prohibited.
    a. Cisapride, dihydroergotamine, ergonovine, ergotamine, methylergonovine, midazolam, pimozide, triazolam or any other major CYP3A4 substrates

IRB Approved
06/24/13

Toxicity Management

Guidelines for grading the severity of adverse experiences are based on DAIDS criteria for grading severity of adverse events from December 2004 (Appendix 1).

Decisions to temporarily withhold therapy because of an adverse experience will be reviewed on a case-by-case basis by the investigator. The investigator should consider temporarily withholding therapy if the severity of the adverse experience is Grade 3 or above and/or if clinically indicated. The decision to interrupt therapy should take into account the patient's baseline laboratory values and any concomitant medications that could be contributory. At the discretion of the investigator, therapy may generally be reinstated when laboratory abnormalities or clinical adverse events return to near normal baseline values.

If after re-initiation of study therapy, there is a recurrence of the laboratory abnormality or clinical adverse event, consideration should be given to permanently discontinuing all study therapy. In general, when a clinical or laboratory adverse event occurs which requires interruption of study therapy, study drug should be interrupted to avoid having a patient receive suboptimal therapy which may predispose them to the development of resistance. In general, study medication should be restarted at full dose concomitantly. Whenever study drug is interrupted due to a study drug related adverse experience, the interruption should be reported on the case report form.

3.2.2 Diet

Patients can take Raltegravir without regard to food consumption. All other antiretroviral drugs in the study will be taken with food.

3.2.3 Procedures

The investigator will follow the patients according to the standard of care. Patients will in addition undergo all the study visits and procedures indicated in the flow chart. Assessment of serious adverse experiences will be made at these visits. All serious adverse events must be immediately reported to the Principal Investigator. In addition, adverse experiences that are drug related and result in Grade 3 or above laboratory toxicity, lead to treatment interruption, or discontinuations (with the reasons for discontinuation) must be reported to the Principal Investigator. Laboratory assays are to be evaluated by the investigator's local laboratory.

Any patient who becomes pregnant during the course of the study must immediately be discontinued from all study medication. All pregnancies must be reported immediately to the Principal Investigator and must be followed to the completion/termination of the pregnancy. The outcome of all pregnancies must be reported to the Principal Investigator.

3.2.3.1 Informed consent

The investigator must obtain documented consent from each potential patient in biomedical research or when an investigational drug is administered to patients in a clinical study, prior to any study related procedures being performed.

IRB Approved
06/24/13

Consent must be documented by the patient's dated signature on a Consent Form along with the dated signature of the person conducting the consent discussion. A copy of the signed and dated consent form should be given to the patient before participating in the trial.

3.2.3.2 Assignment of Baseline Number

For identification purposes during the screening, each patient will be assigned a unique baseline number. He/she will then be randomly assigned to one of the two study groups.

Patients who are HIV seropositive and are antiretroviral naïve (defined as having previously received no antiretroviral therapy or fewer than 7 days of therapy) will be considered for enrollment in this study. The investigator shall discuss with each patient the nature of the study, its requirements, and its restrictions. Written informed consent must be obtained prior to performing any study-specific procedures. Laboratory safely assessment will be performed per study flow chart (Section 1.6). Female patients of childbearing potential will have a pregnancy test performed prior to the study start date. Women who are found to be pregnant will be excluded from the study.

All patients will be asked about prior use of antiretroviral agents and the length of time on therapy with these agents AIDS defining illness, hepatitis B and C history, and genotype and phenotype data. This information will be recorded on the appropriate case report form. If prior genotypic or phenotypic antiretroviral resistance testing is not available, testing must be performed at screening.

3.2.3.3 Allocation

Eighty (80) patients meeting the eligibility requirements will be randomized on a 1:1 basis to one of the two treatment groups:

1. Group A: will receive Raltegravir + Ritonavir-boosted Darunavir
2. Group B: will receive Tenofovir + Emtricitabine + Ritonavir-boosted Darunavir

Patients will be provided with medications according to their treatment groups and will be instructed to take them as previously described. The patients will also be instructed on the procedure for reporting of adverse experiences.

3.2.3.4 Treatment/Evaluation/Follow-up

The investigator will follow the patients according to standard of care.

Laboratory assessments (including HIV RNA and CD4 Cell Count) will be performed locally at the Screen and at all time-points indicated in the study Flow Chart.

Measurement of serum free insulin.

Serum free insulin levels will be measured by radioimmunoassay, using a double antibody technique (Coat-A-Count; Diagnostic Products Corp., Los Angeles, CA). Results will be reported at μU/ml. Insulin resistance will be evaluated by calculation of the homeostasis model assessment (HOMA-IR) calculated from fasting plasma glucose (FPG) and serum free insulin (SFI) with the formula HOMA-IR = SFI in mU/l X FPG in mg/ dl/405.

IRB Approved
06/24/13

In Vivo Lipoprotein Analysis.

Lipoprotein subtype analysis. At baseline and every 16 weeks 2 serum aliquots each with a fill volume of 1 mL will be drawn from the patient after a 12 hours fast per standard blood draw procedures. These samples will be coded with no patient identifiers and frozen and shipped to Pacific Biometrics, Inc. (Seattle, WA) on dry ice. Only the investigators at the VA North Texas Health Care system will be able to link the coded samples to the patient enrolled.

LDL subtypes (LDL1, LDL2a, LDL2b, LDL3, LDL4, LDL5, LDL6, LDL7) and HDL subtypes will be measured using polyacrylamide gradient gel electrophoresis (Berkeley HeartLab, Inc.). Apolipoproteins A-I, A-II, B, C-I, C-II and E will be measured by Immunoturbidimetric assay.

DEXA Scans. Whole body DEXA (Dual Energy X-ray Absorptiometry) scans with 3 bony sites will be done at the VA North Texas Health Care System at baseline and at week 48 of therapy to assess any changes in percent body fat, lean body mass, and bone density.

At each visit, assessment of serious adverse experiences and drug related adverse experiences that result in Grade 3 or above laboratory toxicity, lead to treatment interruption, or discontinuation, and pregnancy test for women of childbearing potential must be performed. Additional recommended procedures are listed on the study flow chart (Section 1.6).

At each visit, the investigator/study coordinator will ascertain the patient's drug compliance, tolerance to the drugs and clinical response prior to providing additional drug supply to last until the next clinic visit. Empty bottles must be returned by the patient to the investigator and recorded in the drug accountability log at the site. All unused study medication and empty bottles will be returned to the Principal Investigator.

All patients that discontinue from the study early must return to the clinic during the follow-up prior for completion of safety assessment and pregnancy test for women of childbearing potential. Additional recommended procedures are listed on the study flow chart.

3.2.3.5 Discontinuation/Withdrawal from Study

Any patient who becomes pregnant during the course of the study must immediately be discontinued from the study. All pregnancies must be reported immediately to the Principal Investigator and must be followed to the completion/termination of the pregnancy. The outcome of the pregnancies must be reported to the Principal Investigator.

Patients may discontinue study therapy:

1. If medically appropriate due to potential clinical and/or laboratory adverse experiences. Discontinuation from the study due to an adverse experience is considered a study-specific adverse event and must be recorded on the appropriate case report form.

IRB Approved
06/24/13

2. If the patient or investigator believes it to be in the patient’s best interest to discontinue.

All patients who discontinue from the study must be reported promptly to one of the Principal Investigator. As soon as possible after discontinuation of all study therapy, patients will have withdrawal visit as noted in the study flow chart.

Study/patients may withdraw at any time or be dropped from the study at the discretion of the investigator should any untoward effects occur. In addition, a subject/patient may be withdrawn by the investigator if he/she violates the study plan or for administrative and/or other safety reasons. The investigator or study coordinator must notify the Primary Investigator immediately when a subject/patient has been discontinued/withdrawn due to an adverse experience (telephone or FAX). When a subject/patient discontinues/withdraws prior to study completion, all applicable activities scheduled for the final study visit should be performed at the time of discontinuation. Any adverse experiences which are present at the time of discontinuation/withdrawal should be followed in accordance with the safety requirements outlined in section 3.3 SAFETY MEASUREMENTS –

3.3 Safety Measurements

3.3.1 Clinical and Laboratory Measurements for Safety

Safety data of serious adverse experiences and drug-related adverse experiences that result in Grade 3 or above laboratory toxicity, lead to treatment interruption, or discontinuation will be collected at each visit. It is recommended that the patient be followed for laboratory safety assessment according to the standard of care. Note that laboratory assays are to be evaluated by the investigator’s local laboratory. The laboratory safety assessments should include a chemistry evaluation with serum creatinine, liver function tests, and a CBC. Additional laboratory assessments should be performed per standard of care at the investigator’s discretion.

In case of grade 3 or 4 laboratory abnormalities, it is recommended to retest within 48 hours for confirmation.

3.3.2 Definition and Classification of Adverse Events

3.3.2.1 Adverse Event

An adverse event is any untoward medical occurrence in a clinical study subject administered a pharmaceutical product. An adverse event does not necessarily have a causal relationship with the treatment. An adverse event can therefore be any unfavorable and unintended sign (including an abnormal finding), symptom, or disease temporally associated with the use of a medicinal (investigational) product, whether or not related to the medicinal (investigational) product. (Definition per International Conference on Harmonisation [ICH])

This includes any occurrence that is new in onset or aggravated in severity or frequency from the baseline condition, or abnormal results of diagnostic procedures, including

IRB Approved
06/24/13

laboratory test abnormalities.

Note: Adverse events can occur from the signing of the informed consent onwards.

3.3.2.2 Serious Adverse Event

A serious adverse event as defined by ICH is any untoward medical occurrence that at any dose meets any of the following conditions:

- results in death
- is life-threatening
  (The subject was at risk of death at the time of the event. It does not refer to an event that hypothetically might have caused death if it were more severe.)
- requires inpatient hospitalization or prolongation of existing hospitalization
- results in persistent or significant disability/incapacity, or
- is a congenital anomaly/birth defect

Note: Medical and scientific judgment should be exercised in deciding whether expedited reporting is also appropriate in situations other than those listed above. For example, important medical events may not be immediately life threatening or result in death or hospitalization but may jeopardize the subject or may require intervention to prevent one of the outcomes listed in the definition above. Any adverse event is considered a serious adverse event if it is associated with clinical signs or symptoms judged by the investigator to have a significant clinical impact.

- **Unlisted (Unexpected) Adverse Event**

An unlisted adverse event, the nature or severity of which is not consistent with the applicable product information. For an investigational product, the expectedness of an adverse event will be determined by whether or not it is listed in the Investigator's Brochure. For a comparator product with a marketing authorization, the expectedness of an adverse event will be determined by whether or not it is listed in the Summary of Product Characteristics (SmPC).

- **Associated With the Use of the Drug**

An adverse event is considered associated with the use of the drug if the attribution is possible, probable, or very likely by the definitions listed below.

3.3.2.3 Attribution Definitions

- **Not related**
  An adverse event that is not related to the use of the drug.
- **Doubtful**
  An adverse event for which an alternative explanation is more likely, e.g., concomitant drug(s), concomitant disease(s), or the relationship in time suggests that a causal relationship is unlikely.

IRB Approved
06/24/13

- **Possible**
  An adverse event that might be due to the use of the drug. An alternative explanation, e.g., concomitant drug(s), concomitant disease(s), is inconclusive. The relationship in time is reasonable; therefore, the causal relationship cannot be excluded.
- **Probable**
  An adverse event that might be due to the use of the drug. The relationship in time is suggestive (e.g., confirmed by dechallenge). An alternative explanation is less likely, e.g., concomitant drug(s), concomitant disease(s).
- **Very likely**
  An adverse event that is listed as a possible adverse reaction and cannot be reasonably explained by an alternative explanation, e.g., concomitant drug(s), concomitant disease(s). The relationship in time is very suggestive (e.g., it is confirmed by dechallenge and rechallenge).

3.3.3 Recording and Reporting of Adverse Experiences

3.3.3.1 All Adverse Events

All adverse events will be documented from the time a signed and dated informed consent form is obtained until completion of the last study-related procedure. Those meeting the definition of serious adverse events must be reported using the Serious Adverse Event Form, including serious adverse events reported to the investigator within 30 days after the subject has completed the last dose of study drug.

All adverse events, regardless of seriousness, severity, or presumed relationship to study therapy, must be documented using medical terminology in the source documents. Whenever possible, diagnoses should be given when signs and symptoms are due to a common etiology (e.g., cough, runny nose, sneezing, sore throat, and head congestion should be reported as “upper respiratory infection”). Investigators must also document their opinion concerning the relationship of the adverse event to study therapy. All measures required for adverse event management must be recorded in the source document.

3.3.3.2 Serious Adverse Events

All serious adverse events occurring during clinical studies will be reported to the appropriate Merck and Tibotec Therapeutics Clinical Affairs contact persons by investigational staff within 24 hours of their knowledge of the event.

Information regarding serious adverse events will be reported to the Institutional Review Board and transmitted to Tibotec Therapeutics Clinical Affairs using the Serious Adverse Event Form, which must be signed by the Primary Investigator. The initial report of a serious adverse event may be made by facsimile (fax to Merck and to Tibotec Therapeutics Clinical Affairs 908-541-4789).

IRB Approved
06/24/13

All serious adverse events that have not resolved by the end of the study, or that have not resolved upon discontinuation of the subject's participation in the study, must be followed until any of the following occurs:

– the event resolves
– the event stabilizes
– the event returns to baseline, if a baseline value is available
– the event can be attributed to agents other than the study drug or to factors unrelated to study conduct
– when it becomes unlikely that any additional information can be obtained (subject or health care practitioner refusal to provide additional information, lost to follow-up after demonstration of due diligence with follow-up efforts)

The cause of death of a subject in a clinical study, whether or not the event is expected or associated with the investigational agent, is considered a serious adverse event. Any event requiring hospitalization (or prolongation of hospitalization) that occurs during the course of a subject's participation in a clinical study must be reported as a serious adverse event, except hospitalizations for:

- social reasons in absence of an adverse event
- surgery or procedure planned before entry into the study

3.3.3.3 Pregnancies

Subject pregnancy must be reported by the investigational staff within 1 working day of their knowledge of the event using the Pregnancy Report form. Any subject who becomes pregnant during the study must be promptly withdrawn from the study. Follow-up information regarding the outcome of the pregnancy and any postnatal sequelae in the infant will be required.

Because the study drug may have an effect on sperm, pregnancies in partners of male subjects included in the study will be reported by the investigational staff within 1 working day of their knowledge of the event using the Pregnancy Report form.

3.4 Data Analysis

The primary analysis of efficacy and safety will be based upon the All Patients As Treated (APaT) approach which includes all patients who received one or more doses of the test drug therapy.

The primary endpoint is the comparison of the efficacy of the test regimen (Raltegravir + Darunavir) to Tenofovir + Emtricitabine + Darunavir. The mean effects of the test regimen (Raltegravir + Darunavir) will be compared to comparator regimen.
The analysis will be time to loss of virologic response (TLOVR) [12]. This analysis defines responders as subjects maintaining a minimum of two sequential HIV viral load <50 c/mL through the scheduled visits without intervening replicated rebounds or treatment discontinuations.

IRB Approved
06/24/13

Logistic regression analysis will be used to analyze outcome data to determine the difference between the two regimens. With 40 patients enrolled in each arm, the study has 80% power to rule out a 15% difference in efficacy between the two regimens. This assumes a true response rate of 85% for both treatment groups.

The reasons for failure at week 48 should be the primary reason for the earliest treatment failure. For any visit, subjects who have achieved confirmed virologic success (two consecutive visits with HIV viral load < 50 copies/mL) will be considered successes. All others should be classified into the other appropriate categories listed in Table 2. All events occurring in the window should be considered events for that visit.

All patients who take study medication will be included in the analysis of safety and tolerability. Only those adverse experiences that occur while on study therapy, or within 14 days after discontinuation of study therapy will be included in the analysis.

For assessment of safety and tolerability, counts and percentages of patients with clinical or laboratory adverse experiences of the following type will be tabulated (1) serious adverse experience, (2) drug-related adverse experiences that result in grade 3 or above laboratory toxicity; (3) drug related adverse experiences that lead to treatment interruption; (4) drug related adverse experiences leading to discontinuation.

Table 2. Display of 48-week efficacy outcome as calculated using the FDA suggested algorithm [12]

**Outcome at 48 weeks**	**Raltegravir + Darunavir N (%)**	**Tenofovir + Emtricitabine + Darunavir N (%)**
**VL <50**	xx (xx%)	xx (xx%)
**VL > 50 (Confirmed)**<br>**Rebound**<br>**Never suppressed through week 48**<br>**Drug change due to virologic failure**	xx (xx%)	xx (xx%)
**Death**	xx (xx%)	xx (xx%)
**Drug change or discontinuation due to adverse events**	xx (xx%)	xx (xx%)
**Drug change or discontinuation due to other reasons**	xx (xx%)	xx (xx%)
**Consent withdrawn**	xx (xx%)	xx (xx%)
**Loss to follow-up**	xx (xx%)	xx (xx%)
**Nonadherence**	xx (xx%)	xx (xx%)
**Pregnancy**	xx (xx%)	xx (xx%)
**Major Protocol violation**	xx (xx%)	xx (xx%)
**Other***	xx (xx%)	xx (xx%)

*Subjects who were never suppressed but were loss to follow-up or introduced new drug therapy before week 48 will be classified into drug change or discontinuation categories.

IRB Approved
06/24/13

3.4.1 Data Monitoring Plan

This study will primarily be conducted at the Dallas VA Medical Center, with participation at Parkland Health & Hospital System as a sub-site. Study staff at the sub-site will recruit and enroll patients who meet the inclusion criteria, and provide patient treatment as outlined in the Study Flow Chart of this protocol (with the exception of DEXA scans, which will be performed at the Dallas VA Medical Center).

The standard laboratory testing will be performed under the same standards for all sites participating in the study. The DAIDS guidelines and criteria for grading toxicities will be followed in all sites. DEXA scans for patients enrolled in all sites will be performed only at the Dallas VA Medical Center. Case report forms (CRF) will be provided for each subject at all sites in order to collect uniform data.

The principal investigator at the Dallas VA (Dr. Roger Bedimo) is also the principal investigator at Parkland Hospital and will be responsible for data security and uniformity. The principal investigator will perform the following duties at all sites: oversee coordination of the study, review all AEs and SAEs, review labs for safety and monitoring purposes, and oversee patient follow-up on a weekly basis.

3.5 Drug Accountability

Table 3: Child-Pugh classification of severity of liver disease

Parameter	Points assigned		
	1	2	3
Ascites	Absent	Slight	Moderate
Bilirubin, mg/dL	<2	2-3	>3
Albumin, g/dL	>3.5	2.8-3.5	<2.8
Prothrombin time			
Seconds over control	<4	4-6	>6
INR	<1.7	1.7-2.3	>2.3
Encephalopathy	None	Grade 1-2	Grade 3-4

Modified Child-Pugh classification of the severity of liver disease according to the degree of ascites, the plasma concentrations of bilirubin and albumin, the prothrombin time, and the degree of encephalopathy. A total score of 5-6 is considered grade A (well-compensated disease); 7-9 is grade B (significant functional compromise); and 10-15 is grade C (decompensated disease). These grades correlate with one- and two-year patient survival: grade A - 100 and 85 percent; grade B - 80 and 60 percent; and grade C - 45 and 35 percent.

IRB Approved
06/24/13

References:

1. Molto, J., et al., *Lopinavir/ritonavir monotherapy as a simplification strategy in routine clinical practice.* J Antimicrob Chemother, 2007.
2. Arribas, J.R., et al., *Lopinavir/ritonavir as single-drug therapy for maintenance of HIV-1 viral suppression: 48-week results of a randomized, controlled, open-label, proof-of-concept pilot clinical trial (OK Study).* J Acquir Immune Defic Syndr, 2005. **40**(3): p. 280-7.
3. Tebas, P., et al., *Switching to a Protease Inhibitor-Containing, Nucleoside-Sparing Regimen (Lopinavir/Ritonavir Plus Efavirenz) Increases Limb Fat But Raises Serum Lipid Levels: Results of a Prospective Randomized Trial (AIDS Clinical Trial Group 5125s).* J Acquir Immune Defic Syndr, 2007. **45**(2): p. 193-200.
4. Nolan, D., P. Reiss, and S. Mallal, *Adverse effects of antiretroviral therapy for HIV infection: a review of selected topics.* Expert Opin Drug Saf, 2005. **4**(2): p. 201-18.
5. Fischl, M.A., et al., *Randomized open-label trial of two simplified, class-sparing regimens following a first suppressive three or four-drug regimen.* Aids, 2007. **21**(3): p. 325-33.
6. Allavena, C., et al., *Efficacy and tolerability of a nucleoside reverse transcriptase inhibitor-sparing combination of lopinavir/ritonavir and efavirenz in HIV-1-infected patients.* J Acquir Immune Defic Syndr, 2005. **39**(3): p. 300-6.
7. Wheeler, W., et al. *Antiretroviral Drug-resistance Mutations and Subtypes in Drug-naïve Persons Newly Diagnosed with HIV-1 Infection, US, March 2003 to October 2006* in *14th Conference on Retroviruses and Opportunistic Infections*. 2007. Los Angeles, California.
8. Markowitz, M., et al. *Potent Antiretroviral Effect of MK-0518, a Novel HIV-1 Integrase Inhibitor, as part of Combination ART in Treatment -Naïve HIV-1 infected Patients* in *XVI International AIDS Conference*. 2006. Toronto, Canada.
9. Markowitz, M., et al., *Antiretroviral activity, pharmacokinetics, and tolerability of MK-0518, a novel inhibitor of HIV-1 integrase, dosed as monotherapy for 10 days in treatment-naive HIV-1-infected individuals.* J Acquir Immune Defic Syndr, 2006. **43**(5): p. 509-15.
10. Cooper, D., et al. *Results of BENCHMRK-1, a Phase III Study Evaluating the Efficacy and Safety of MK-0518, a Novel HIV-1 Integrase Inhibitor, in Patients with Triple-class Resistant Virus*. in *14th Conference on Retroviruses and Opportunistic Infections*. 2007. Los Angeles.
11. Steigbigel, R.T., et al. *Results of BENCHMRK-2, a Phase III Study Evaluating the Efficacy and Safety of MK-0518, a Novel HIV-1 Integrase Inhibitor, in Patients with Triple-class Resistant Virus*. in *14th Conference on Retroviruses and Opportunistic Infections*. 2007. Los Angeles.
12. *Guidance for industry: antiretroviral drugs using plasma HIV RNA measurements -- clinical considerations for accelerated and traditional approval. US Depart of Health and Human Services, Food and Drug Administration, Center for Drug Evaluation and Research; October 2002.*
13. *Efficacy and safety of darunavir-ritonavir at week 48 in treatment-experienced patients with HIV-1 infection in POWER 1 and 2: a pooled subgroup analysis of*

IRB Approved
06/24/13

data from two randomised trials. Lancet. 2007 Apr 7;369(9568):1169-78. Erratum in: Lancet. 2008 Jan 12;371(9607):116.

14. *DeJesus E, Ortiz R, Khanlou H, et al. Efficacy and safety of darunavir/ritonavir versus lopinavir/ritonavir in ARV treatment-naive HIV-1-infected patients at week 48: ARTEMIS (TMC114-C211). 47th Interscience Conference on Antimicrobial Agents and Chemotherapy. September 17-20, 2007. Chicago. Abstract H-718b.*
15. *Sekar V et al. 15th Conference on Retroviruses and Opportunistic Infections. February 3-6, 2008. Boston. Abstract 769)*

IRB Approved
06/24/13
